# Integrative Multi-Omics Analysis Reveals the Characteristic Metabolic Signature of Glioma and Enables Plasma-Based Liquid Biopsy

**DOI:** 10.34133/research.1199

**Published:** 2026-03-23

**Authors:** Yixiao Jiang, Yufei Lan, Yifeng Wang, Sui Chen, Yixiong Shen, Shiyao Chu, Yaoyuan Dong, Lei Li, Huan Zhang, Zhijie Lu, Yuankai Wang, Jiankun Lu, Xiaoman Li, Feiyunduo Hao, Qu Yue, Hongbo Guo

**Affiliations:** ^1^ Neurosurgery Center, The National Key Clinical Specialty, The Engineering Research Center of Diagnostic and Therapeutic Technology and Devices for Cerebrovascular Diseases in Ministry of Education, Guangdong Provincial Key Laboratory on Brain Function Repair and Regeneration, Zhujiang Hospital Institute for Brain Science and Intelligence, Zhujiang Hospital, Southern Medical University, Guangzhou 510282, China.; ^2^ Department of Functional Neurosurgery, Zhujiang Hospital, Southern Medical University, Guangzhou 510282, China.

## Abstract

Liquid biopsy strategies for glioma leveraging metabolic features remain inadequately investigated. Herein, we performed liquid chromatography-mass spectrometry-based metabolomic and proteomic analyses on 189 tissue samples from 122 adult glioma patients, and nuclear magnetic resonance-based targeted metabolomic profiling on plasma samples from 430 participants encompassing 82 adult glioma patients, 53 pediatric primary brain tumor patients, 80 pancreatic cancer patients, and 215 nontumor controls. The results demonstrate that aberrations in “Alanine, aspartate, and glutamate metabolism” and “tricarboxylic acid (TCA) cycle” pathways are ubiquitous across subtypes and progression of glioma. Notably, these signatures could be captured in plasma, thereby reflecting shared metabolic features between tumor tissues and circulation. Based on these findings, we developed a liquid biopsy model comprising 7 plasma metabolites (including creatine, lactic acid, succinic acid, N,N-dimethylglycine, 2-oxoglutaric acid, acetic acid, and glutamic acid). This model achieved high diagnostic accuracy in independent test sets (area under the curve = 0.964 for adult glioma set; and 0.925 for pediatric primary brain tumor set). Meanwhile, the model exhibited a higher sensitivity of 0.885 for glioma compared to 0.800 for pancreatic cancer, providing evidence to support the tumor selectivity of the model. Together, we present a plasma-based metabolomic classifier that faithfully mirrors the core metabolic reprogramming of glioma and can serve as a readily available liquid biopsy tool.

## Introduction

Glioma represents the most common primary malignant brain tumor [1,2]. Accurate diagnosis is fundamental for managing glioma patients [3]. Clinical diagnosis of malignant tumors typically relies on imaging, circulating biomarkers, and pathological biopsy [4–6]. However, unlike tumors in other organs, intracranial tumor biopsy carries substantial risks, particularly for lesions located in the brainstem or eloquent areas [7,8]. Noninvasive neuroimaging remains the primary preoperative modality for glioma diagnosis and lesion delineation. Its diagnostic accuracy in glioma is only approximately 70% due to limitations in precision and resolution of imaging technologies [9]. This limitation is especially pronounced in early-stage gliomas with small tumor volumes, underscoring the urgent need to develop liquid biopsy techniques [10,11].

Currently, there are no approved circulating biomarkers for clinical use in glioma. The blood–brain barrier (BBB) severely restricts the detectable levels of genetic mutations derived from intracranial tumors in the blood circulation [12,13]. Liquid biopsy approaches such as analyses of circulating tumor cells (CTCs) or cell-free DNA (cfDNA) have not yielded satisfactory results [14,15]. Metabolic reprogramming is one of the most prominent features of glioma, and pathological subtypes along with malignancy grades can be discriminated using metabolomic profiling [16,17]. Metabolic alterations can precede clinical diagnosis by several years, implying that reprogramming is an early driver of gliomagenesis [18]. Furthermore, owing to their small molecular size, metabolites can freely pass through the compatible pores of cerebral microvascular endothelial cells [19]. Emerging evidence indicates that serum metabolic profiles may capture key metabolic information of intracranial tumors, representing a promising alternative for liquid biopsy [3,9].

Despite this promise, developing a glioma liquid biopsy strategy based on peripheral blood metabolomics faces challenges. The most notable is the high heterogeneity of glioma, where metabolic reprogramming characteristics vary vastly among different pathological subtypes [16,20]. Studies have also revealed substantial heterogeneity within different regions of the same glioma [21]. A critical unresolved issue is how to effectively identify common metabolic alteration features during the transformation from glial cells to invasive malignant cells with distinct biological behaviors. Secondly, tumor progression often induces marked systemic metabolic shifts to maintain tumor microenvironment homeostasis and meet the nutritional demands of rapid proliferation [22–24]. However, the relationship between these systemic metabolic changes and localized tumor tissue metabolic reprogramming in glioma patients remains largely unexplored.

In this study, we aim to investigate the shared metabolic alterations between glioma tissue and plasma and to identify plasma biomarkers that accurately reflect tumor metabolic reprogramming, thereby providing a theoretical foundation for developing a metabolomics-based liquid biopsy strategy for glioma. We employed integrated multi-omics analyses to reveal tumor-specific metabolic markers during glioma progression. Based on shared metabolic reprogramming features in plasma and local tumor tissue, we developed and validated a nuclear magnetic resonance (NMR)-based liquid biopsy strategy for glioma. Our results demonstrated that this metabolomics approach faithfully recapitulated glioma-specific metabolic reprogramming and achieved sufficient clinical sensitivity and specificity for both adult and pediatric patients, suggesting its potential for glioma screening and auxiliary diagnosis.

## Results

### Study design and participant clinical information

**Fig. 1.**
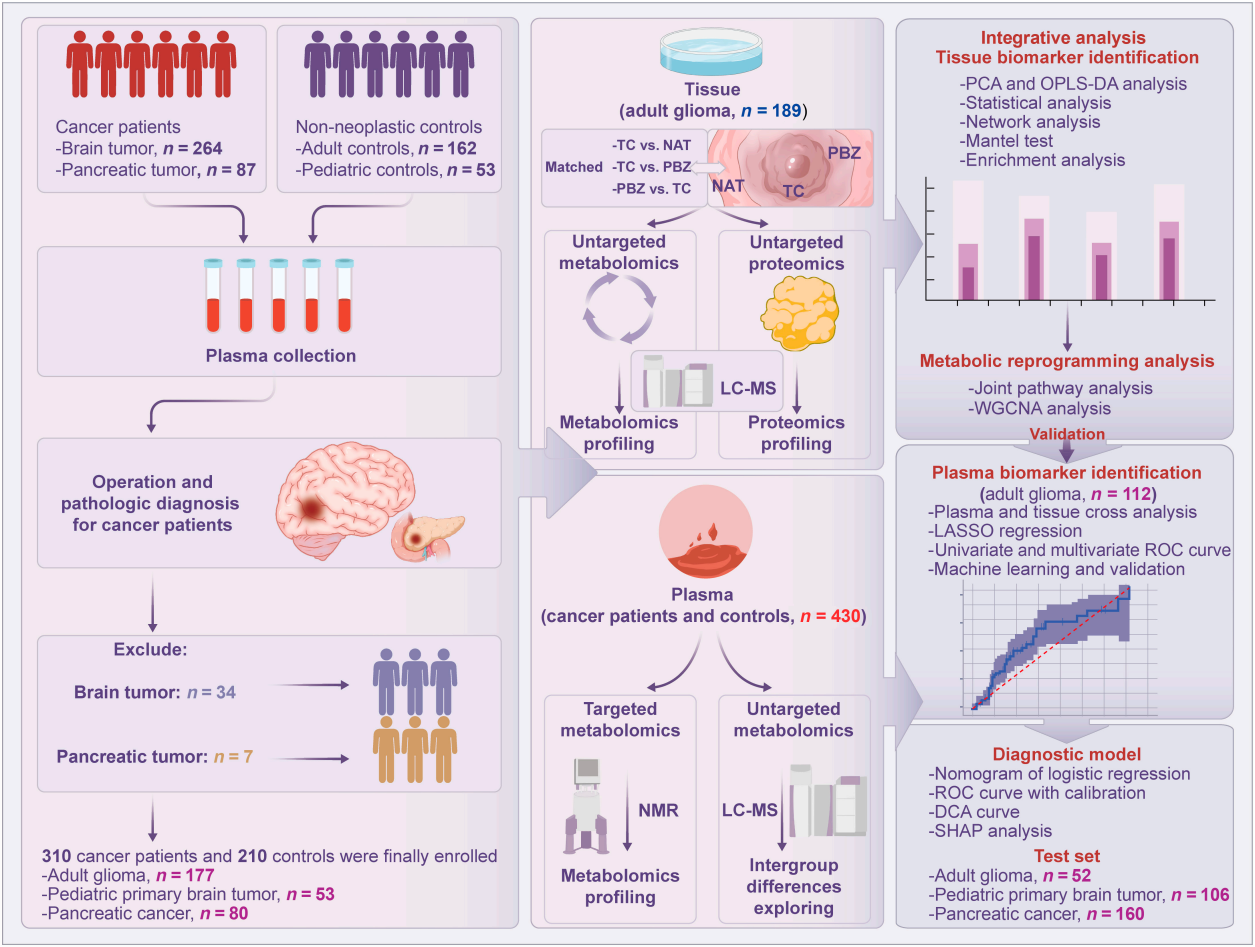
Schematic diagram of overall study design. A total of 430 plasma samples from patients or healthy volunteers, along with tissue samples from 122 adult glioma patients, were included in this study. Untargeted metabolomics and proteomics profiling were conducted on the tissue samples using liquid chromatography-mass spectrometry (LC-MS). Targeted metabolomics analysis was performed on the plasma samples. Subsequently, biomarker identification, diagnostic model development, and model validation were carried out. NMR, nuclear magnetic resonance; ROC, receiver operating characteristic; DCA, decision curve analysis; SHAP, Shapley additive explanation.

A total of 566 participants, comprising glioma patients, pancreatic cancer patients, and nontumor controls, were enrolled at Zhujiang Hospital, Guangzhou, China, while 34 patients preoperatively diagnosed with glioma and 7 with pancreatic cancer who were excluded for postoperative pathology did not meet the pathological diagnostic criteria. The overall study design and sample sizes are summarized in Fig. 1.

For the investigation of metabolic reprogramming in tumor tissues, 189 tissue samples from 122 adult glioma patients were analyzed. Consistent with previous studies [21,25,26], the tumor core (TC) was defined as the region showing marked enhancement on T1-weighted contrast-enhanced magnetic resonance imaging (MRI). The peritumoral brain zone (PBZ) was considered as the tissue surrounding the T1-weighted contrast-enhancing lesion within a distance of 1 cm, representing as the tumor-brain interface. Normal adjacent tissue (NAT) referred to tissue located more than 1 cm from the enhancing border. During surgery, NAT samples were generally obtained from superficial nontumorous brain tissue resected as part of the necessary surgical access, ensuring no additional risk to the patients. The clinical and demographic characteristics of the adult glioma participants who provided valid tissue samples are summarized in Table S1, with detailed clinical information provided in Data file S1.

In the plasma metabolomics analysis and diagnostic model development, 430 participants, including tumor patients and nontumor controls, were assigned to a training set and 3 test sets. The training set consisted of 56 adult glioma patients and 56 adult nontumor controls recruited between January 2022 and March 2023. The adult glioma test set included 26 adult glioma patients and 26 adult health controls enrolled from March 2023 to August 2025. The pediatric primary brain tumor test set comprised 53 pediatric patients with primary intracranial tumors and 53 pediatric nontumor controls. To measure the tumor specificity of the model, we also recruited an adult pancreatic cancer test set that consisted of 80 patients with pancreatic ductal adenocarcinoma and 80 adult nontumor controls. The clinicopathological and demographic characteristics of participants in the training set and test sets are detailed in Tables S2 to S4, while individual clinical data for each tumor patient are provided in Data file S2.

### Metabolomic landscape of glioma tissues

**Fig. 2.**
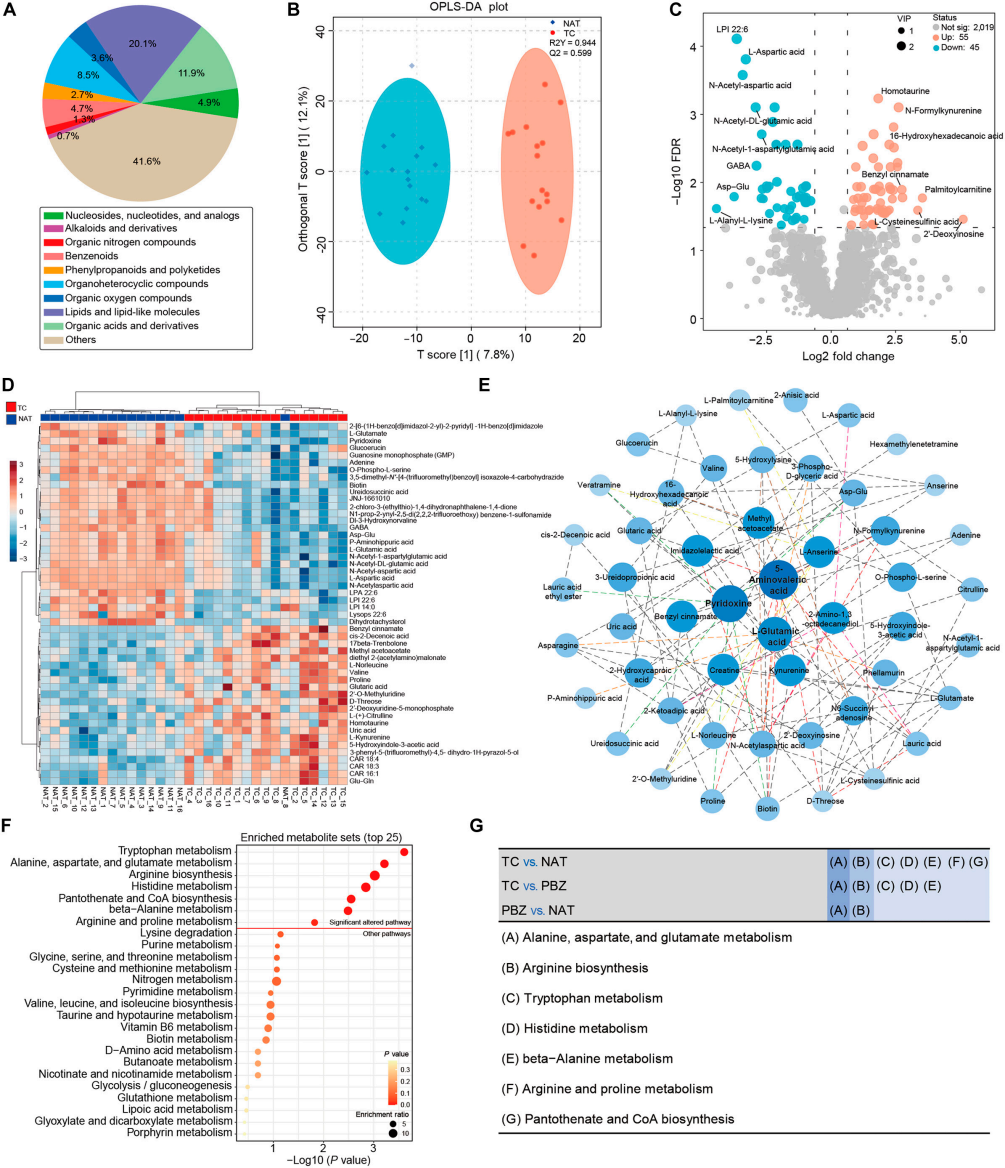
Tissue metabolomic landscape of adult glioma based on liquid chromatography-mass spectrometry-based metabolomics. (A) Pie chart showing the distribution of metabolite abundances across different groups, with colors representing various metabolite categories. (B) Orthogonal partial least squares-discriminant analysis (OPLS-DA) score plot for metabolomics in matched tumor core (TC) and normal adjacent tissue (NAT) tissues, with 95% confidence intervals annotated. (C) Volcano plot displaying differentially abundant metabolites between TC and NAT, including variable importance in projection (VIP) values. Significance thresholds: false discovery rate (FDR) < 0.05, fold change (FC) > 1.5 or < 0.667, VIP > 1.0. The Benjamini–Hochberg FDR method was used to correct for multiple comparisons. Differentially abundant metabolites are shown as red dots (up-regulated) or blue dots (down-regulated), while gray dots indicate no significant difference. (D) Unsupervised hierarchical clustering analysis heatmap of metabolites comparing TCs with adjacent normal brain tissues. Red indicates increased metabolite levels, while blue indicates decreased metabolite levels. (E) Debiased sparse partial correlation network analysis of differentially abundant metabolites. Each node represents a metabolite molecule, with darker colors indicating stronger associations with other metabolites, especially those closer to the center. (F) Top 25 most significantly altered metabolic pathways in glioma TC tissues, with red lines delineating metabolic pathways showing significant perturbations (*P* < 0.05). (G) Statistical analysis of major metabolic pathway dysregulation (*P* < 0.05) during different stages of glioma progression.

Untargeted metabolomic analysis of glioma TC, PBZ, and NAT samples was performed using a liquid chromatography-mass spectrometry (LC-MS) platform. After data filtering, 2,119 metabolites confidently identified at Level 2 or above (Metabolomics Standards Initiative [27,28]) were detected and annotated, comprising 1,345 metabolites in positive-ion mode and 774 metabolites in negative-ion mode. Quality control (QC) samples were utilized for correlation analysis (Fig. S1A), and principal component analysis (PCA) was conducted to evaluate data quality and global metabolic variations (Fig. S1B). The chemical classification of all identified metabolites is summarized in Fig. 2A.

For multivariate analysis, orthogonal partial least squares-discriminant analysis (OPLS-DA) was performed (Fig. 2B). Model validity was confirmed through permutation test (Fig. S1C). Metabolites significantly contributing to group separation were identified based on variable importance in projection (VIP) scores (Fig. S1D). A volcano plot was used to visualize significantly up-regulated and down-regulated metabolites in TC compared to NAT tissues, with 100 metabolites meeting the screening criteria (fold change [FC] > 1.5 or < 0.667, VIP > 1.0, false discovery rate [FDR] < 0.05). Among these, 55 metabolites, including homoserine, 2′-deoxyinosine, and 16-hydroxyhexadecanoic acid, were up-regulated in tumor tissues, while 45 metabolites were down-regulated, including L-aspartic acid, L-glutamic acid, γ-aminobutyric acid, and Asp–Glu (Fig. 2C). Hierarchical clustering analysis (HCA) of the top 50 altered metabolites effectively distinguished TC from NAT tissues (Fig. 2D).

The debiased sparse partial correlation network revealed significant associative relationships among the differential metabolites (Fig. 2E). Topological analysis indicated that L-glutamic acid, pyridoxine, and 5-aminovaleric acid occupied central positions, exhibiting the most intensive interactions with metabolites involved in nucleotide, lipid, amino acid, and energy metabolism, suggesting their potential role as core molecules in glioma metabolic alterations. Furthermore, Kyoto Encyclopedia of Genes and Genomes (KEGG) pathway enrichment analysis highlighted significant disturbances in metabolic pathways within glioma tissues, including “Tryptophan metabolism”, “Alanine, aspartate, and glutamate metabolism”, and pathways related to arginine metabolism (Fig. 2F). The expression levels of significantly altered metabolites within the “Alanine, aspartate, and glutamate metabolism” pathway across TC and NAT tissues were detailed by violin plots (Fig. S1E).

Comparative analysis of metabolite levels across different spatial regions of glioma tissues identified 57 differential metabolites between TC and PBZ and 104 differential metabolites between PBZ and NAT (Fig. S2A and B). KEGG pathway analysis suggested that “Alanine, aspartate, and glutamate metabolism” and “Arginine biosynthesis” were key metabolic pathways altered during the progression from normal brain tissue to densely tumor cell-populated region (Fig. 2G and Fig. S2C and D). Furthermore, cross-comparison among the 3 tissue types with varying degrees of tumor infiltration revealed 12 common differential metabolites (Fig. S2E). The expression trends and statistical details of these shared differential metabolites are presented in Table S5. Figure S2F illustrates these metabolites, which exhibited consistent expression trends throughout glioma progression, indicating their potential as key metabolic markers in this process.

### Proteomic landscape of glioma tissues

**Fig. 3.**
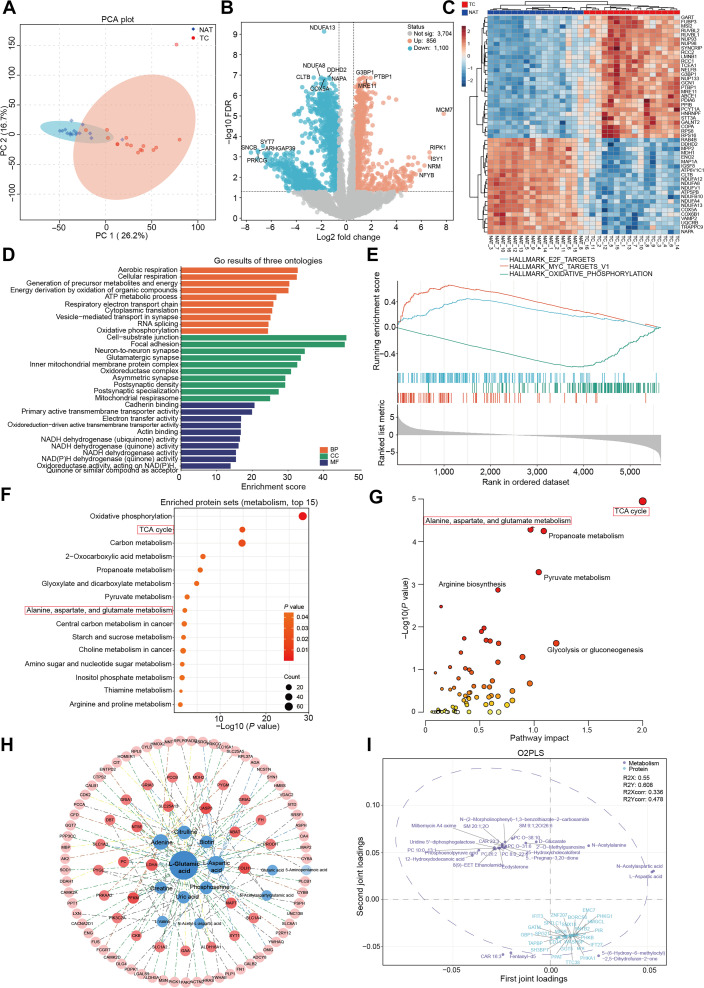
Metabolomic and proteomic integrated analysis identifies key features of metabolic reprogramming in adult glioma. (A) Loading plot of the principal component analysis (PCA) model for liquid chromatography-mass spectrometry-based proteomics in glioma tissues, with 95% confidence intervals annotated. (B) Volcano plot displaying differentially abundant proteins between tumor core and adjacent normal brain tissues. Significance thresholds: false discovery rate (FDR) < 0.05, fold change > 1.5 or < 0.667. Differentially abundant proteins are shown as red dots (up-regulated) or blue dots (down-regulated), while gray dots indicate no significant difference. (C) Heatmap visually displays the top 50 proteins with abnormally high or low expression across different groups. Each colored square corresponds to a relative concentration value, with samples represented in rows and protein names in columns. (D) Gene Ontology (GO) pathway enrichment analysis of differentially expressed proteins between tumor core and adjacent normal brain tissues. The top 10 enriched pathways for Biological Process (BP), Cellular Component (CC), and Molecular Function (MF) are displayed, all showing statistically significant differences (*P* < 0.05). (E) Gene set enrichment analysis illustrates the 3 most significantly perturbed HALLMARK pathways in proteins from glioma core tissues compared to adjacent normal brain tissues. (F) Kyoto Encyclopedia of Genes and Genomes pathway enrichment analysis of differentially abundant proteins between tumor core and adjacent normal brain tissues, with the top 15 metabolism-related pathways illustrated. All pathways shown in the figure exhibit significant statistical differences (*P* < 0.05). (G) Joint pathway analysis of differentially abundant metabolites (from Fig. 2B) and differentially expressed proteins (from Fig. 2G) between tumor core and normal adjacent tissue tissues. This integrated approach identifies metabolic pathways significantly coaltered at both the metabolite and protein levels in glioma. (H) Protein–metabolite interaction network constructed using differentially expressed proteins with Pearson correlation coefficients > 0.7. Each node represents either a differentially expressed protein (red) or a differentially abundant metabolite (blue). Node size is proportional to its eigenvector centrality (reflecting network influence), and edge proximity indicates the strength of correlation, with more strongly correlated nodes positioned closer together. Darker-colored nodes located near the network core represent molecules with stronger associations to other network components. (I) O2PLS-DA loading plot based on differential proteins and metabolites. The top 25 metabolites most influential in distinguishing tumor core tissue are highlighted in purple, while the top 25 proteins are highlighted in blue, illustrating their combined contribution to the metabolic reprogramming phenotype in glioma core regions. TC, tumor core.

To provide complementary insights at the protein level, we performed data-independent acquisition (DIA) mode-based proteomic analysis using glioma tissues from the same cohort. A total of 216,732 peptides and 11,658 proteins were identified. QC and HCA of the entire proteome are presented in Fig. S3A to E. PCA revealed a clear separation between the TC and NAT groups based on their protein profiles (Fig. 3A and Fig. S3F and G). Univariate analysis identified 856 proteins up-regulated and 1,100 proteins down-regulated in TC compared to NAT (Fig. 3B). Hierarchical clustering of the top 50 most significantly altered proteins effectively distinguished TC from NAT tissues (Fig. 3C).

To elucidate the associated functional alterations, we subjected differentially expressed proteins (DEPs) to Gene Ontology (GO), gene set enrichment analysis (GSEA), and KEGG pathway analyses. GO analysis revealed significant enrichment in 987 biological process terms, 270 cellular component terms, and 184 molecular function terms, indicating extensive and complex alterations at the protein level in glioma. The most significantly altered pathways were associated with energy metabolism, mitochondrial function, oxidoreductase activity, and synaptic function (Fig. 3D). GSEA indicated activation of the “E2F and MYC pathways”, alongside significant suppression of “Oxidative phosphorylation” (Fig. 3E). Furthermore, KEGG analysis mapped the DEPs to 126 metabolic pathways. Figure 3F displays the top 15 most significantly enriched metabolism-related pathways, which included well-established pathways such as the “tricarboxylic acid (TCA) cycle”, “Oxidative phosphorylation”, and “Pyruvate metabolism”. Notably, the “Alanine, aspartate, and glutamate metabolism pathway” was significantly altered at both the metabolite and protein levels. Cnet plots illustrated the expression level of proteins involved in enriched energy and amino acid metabolic pathways (Fig. S4A and B).

Analysis of DEPs across spatially distinct glioma tissues revealed 467 proteins that were significantly altered along the progression trajectory from NAT to TC, suggesting their potential critical roles in gliomagenesis and progression (Fig. S5A and B). Joint pathway analysis, combining the common differential metabolites from Fig. S2E and the common differential proteins from Fig. S5A, demonstrated significant perturbations in key metabolic pathways, including “Alanine, aspartate, and glutamate metabolism” and “TCA cycle” (Fig. S5C). Within the “Alanine, aspartate, and glutamate metabolism pathway”, key enzymes such as N-acetyltransferase 8-like (NAT8L), glutaminase (GLS), glutamic-oxaloacetate transaminase 1 (GOT1), glutamate-ammonia ligase (GLUL), and carbamoyl-phosphate synthetase 2, aspartate transcarbamylase, and dihydroorotase (CAD) exhibited significant expression changes during the spatial progression of glioma. Similarly, key enzymes in the “TCA cycle”, including malate dehydrogenase 1, citrate synthase, isocitrate dehydrogenase (IDH) and succinyl-CoA ligase GDP-forming subunit beta (SUCLG2), showed marked differential expression across the spatial trajectory of glioma development. Figure S5D and E visually summarizes the expression patterns of these proteins throughout the spatial progression of glioma, highlighting their consistent dysregulation. Together, these results confirm that the “Alanine, aspartic acid and glutamic acid metabolism pathway” and “TCA cycle” pathway exhibit persistent abnormalities during the progression of normal brain tissue to the core tissue of tumors at protein level, which are the key metabolic reprogramming mechanisms driving the occurrence and development of gliomas.

To further investigate the protein biomarkers in glioma tissue, we analyzed a cohort of 163 glioblastoma (GBM) samples from The Cancer Genome Atlas database and 207 normal brain tissue samples from the Genotype-Tissue Expression database. Differential expression analysis identified 6,339 differentially expressed genes. By integrating Kaplan–Meier survival analysis performed using the Gene Expression Profiling Interactive Analysis 2 platform, we identified 7 proteins that met the predefined screening criteria (Fig. S6A). Among these, 5 proteins (prolyl 4-hydroxylase subunit beta (P4HB), promyelocytic leukemia protein (PML), protein disulfide isomerase family A member 4 (PDIA4), cathepsin B (CTSB), and procollagen-lysine, 2-oxoglutarate 5-dioxygenase 3 (PLOD3)) were significantly up-regulated in glioma tissues, while acyl-CoA thioesterase 7 and hippocalcin-like 1 were significantly down-regulated (Fig. S6B). The expression trends of these key proteins at the gene level were consistent with our proteomic findings (Fig. S6C). Importantly, Kaplan–Meier survival analysis indicated that high expression of these proteins was associated with poor prognosis in GBM patients (Fig. S6D). These results suggest that these proteins are not only dysregulated during glioma development but also impact patient outcomes, highlighting their potential as biomarkers and therapeutic targets.

### Multi-omics analysis identifies key features of metabolic reprogramming in glioma

To elucidate the core characteristics of metabolic reprogramming in glioma, joint pathway we performed analysis using the 100 differential metabolites from Fig. 2C and the 1,956 DEPs from Fig. 3B, with annotations by KEGG. The results demonstrated significant perturbations in energy metabolism and amino acid metabolism pathways, represented by the “TCA cycle”, and “Alanine, aspartate, and glutamate metabolism” (Fig. 3G and Fig. S7A and B). Subsequently, we constructed an interaction network integrating differential proteins and metabolites from TC versus NAT tissues to identify coregulated nodes. The network analysis, showcasing the metabolites and proteins with the strongest associations, emphasized L-glutamic acid as a central metabolite in glioma metabolic reprogramming, with robust correlations with metabolites, various transport proteins, and enzymes (Fig. 3H). Dysregulation of energy metabolism, as a hallmark of the Warburg effect, has been extensively documented in glioma research [29]. Our study highlights the “Alanine, aspartate, and glutamate metabolism pathway” as being profoundly altered and intricately linked to energy metabolism in glioma metabolic reprogramming. Key metabolites within this pathway, including L-aspartic acid, L-glutamic acid, and γ-aminobutyric acid, were significantly down-regulated in glioma tissues. Furthermore, among the bridging metabolites connecting these 2 metabolism pathways, 2-oxoglutaric acid levels were markedly elevated, while succinic acid levels decreased in tumor tissues. These findings supported the idea that “Alanine, aspartate, and glutamate metabolism” constitutes another core metabolic pathway in glioma reprogramming, closely associated with the Warburg effect, thereby warranting deeper investigation.

To further validate the coordinated variations between the metabolome and proteome in glioma tissues, we applied a 2-way orthogonal partial least squares (O2PLS) model to identify the most strongly correlated metabolites and proteins (Fig. 3I). Integrated analysis of key metabolites and proteins in O2PLS model further confirmed “Alanine, aspartate, and glutamate metabolism” as a pivotal pathway in glioma metabolic reprogramming (Fig. S7C). We employed Mantel tests to assess the correlations between differential metabolites and proteins within the most significantly perturbed amino acid metabolism pathways (Fig. S7D). The analysis revealed that metabolites in the “Alanine, aspartate, and glutamate metabolism” were significantly positively correlated with ACO1, ASS1, and NAT8L, and negatively correlated with GLS. In addition, metabolites in the “Arginine biosynthesis” showed a significant positive correlation with ADSSL1 and negative correlations with ASS1, CAD, GLS, GLUL, ABAT, ADSL, ASRGL1, NAT8L, and PPAT.

### Metabolic hallmarks of adult glioma subtypes

**Fig. 4.**
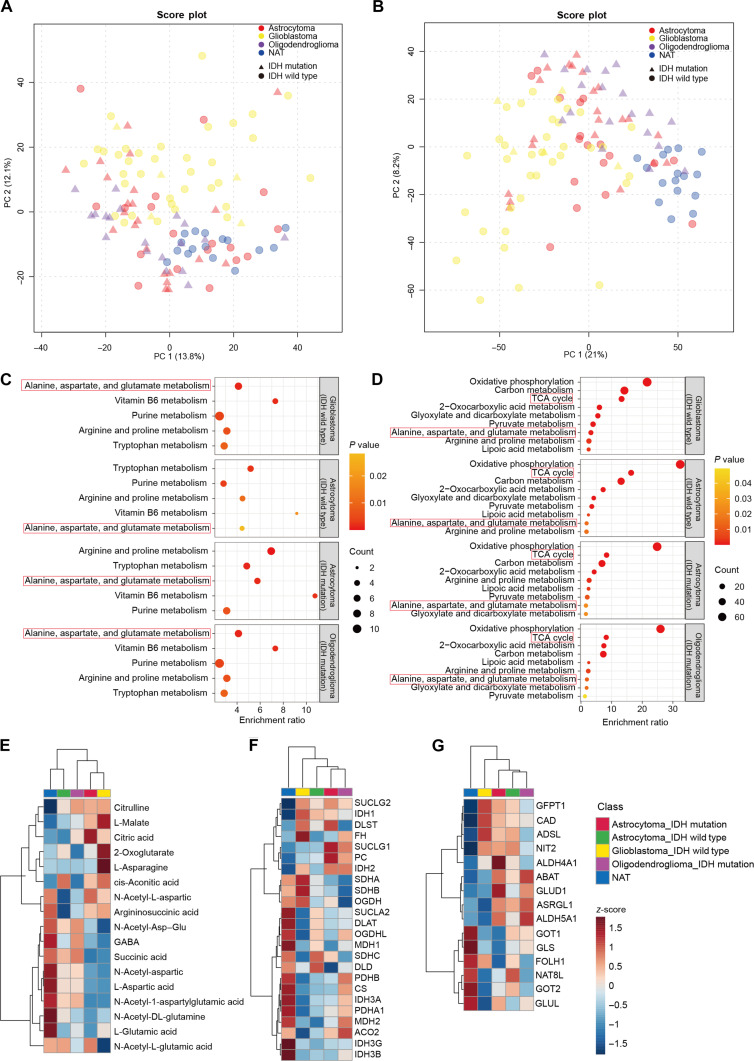
Metabolic characteristics of adult glioma subtypes. (A and B) Principal component analysis of the metabolome (A) and proteome (B) in tumor core and normal adjacent tissue (NAT) across different adult glioma subtypes. Differences between astrocytoma (red, *n* = 40), glioblastoma (yellow, *n* = 40), oligodendroglioma (purple, *n* = 19), and adjacent normal brain tissue (blue, *n* = 16) are illustrated. Isocitrate dehydrogenase (IDH)-wild-type samples and adjacent normal brain tissue samples are represented by dots, while IDH-mutant samples are represented by triangles. Oligodendroglioma samples with IDH wild type were excluded due to insufficient sample size. (C and D) Kyoto Encyclopedia of Genes and Genomes term enrichment analysis of differentially expressed metabolites (C) and proteins (D) (significance threshold: false discovery rate < 0.05; fold change > 1.5 or < 0.667). The figures show the common metabolic pathways in the metabolomic and proteomic analyses of the 4 major adult glioma subtypes. (E) Unsupervised hierarchical clustering analysis heatmap based on normalized *z*-scores displaying the average expression of key metabolites in the “Alanine, aspartate, and glutamate metabolism” and “TCA cycle” across each glioma-subtype tissue sample group and NAT sample group. (F and G) Unsupervised hierarchical clustering analysis heatmap based on normalized *z*-scores displaying the average expression of key proteins in the “TCA cycle” (F) and “Alanine, aspartate, and glutamate metabolism” (G) pathways across each glioma-subtype tissue sample group and NAT sample group.

Compared with other solid tumors, gliomas exhibit more complex subtypes. To identify shared metabolic characteristics among adult glioma subtypes, we analyzed metabolomic and proteomic datasets from 4 major adult glioma subtypes: IDH-wild-type GBM, IDH-wild-type astrocytoma, IDH-mutant astrocytoma, and IDH-mutant oligodendroglioma. PCA plots revealed distinct metabolite and protein expression patterns among different glioma subtypes, with GBM showing greater heterogeneity (Fig. 4A and B).

To determine which metabolic pathways underlie the subtype-specific expression profiles in adult gliomas, we performed KEGG enrichment analysis on differential expression metabolites and proteins of each adult glioma subtype. The results showed that 5 metabolic pathways were enriched in the differential metabolites and 9 in the differential proteins across different glioma subtypes (Fig. S8A and B). Notably, the “Alanine, aspartate, and glutamate metabolism” pathway was significantly enriched in both the metabolomic and proteomic analyses (Fig. 4C). “TCA cycle” pathway was one of the most critically altered metabolic pathways at the proteomic level across different glioma subtypes (Fig. 4D). These findings further emphasize that the perturbations in these pathways represent key metabolic transitions in adult gliomas.

To further explore the differences in metabolite and protein levels within the identified key metabolic pathways across different glioma subtypes, we conducted HCA based on average metabolite and protein expression levels. At the metabolite level, compared to normal brain tissues, energy metabolism intermediates such as L-malate and 2-oxoglutaric acid were elevated in glioma tissues, while amino acids like L-glutamic acid and L-aspartic acid were decreased (Fig. 4E). This phenomenon was more pronounced in IDH-wild-type GBM. At the protein level, energy metabolism-related proteins exhibited significant differences in glioma tissues: SUCLG1/2 and IDH1/2 were significantly increased, whereas SUCLA2 and malate dehydrogenase 1/2 were significantly decreased (Fig. 4F). In addition, proteins involved in amino acid pathways including CAD, GLUD1, GOT1/2, and GLS showed significant differences (Fig. 4G). Together, these data indicate that alterations in the “Alanine, aspartate, and glutamate metabolism” and “TCA cycle” pathways are common metabolic features across different subtypes of adult gliomas, with the most malignant GBM exhibiting more pronounced changes in these aspects.

### WGCNA identifies hub metabolites and proteins in GBM

GBM represents one of the most common adult glioma subtypes, characterized by rapid proliferation and poor prognosis. Our previous findings indicated that GBM exhibits more complex metabolic reprogramming mechanisms compared to other adult glioma subtypes. To further identify hub metabolites and protein modules driving GBM pathogenesis, we performed weighted gene coexpression network analysis (WGCNA) on TC and PBZ tissues from 20 paired samples of IDH-wild-type GBM patients.

**Fig. 5.**
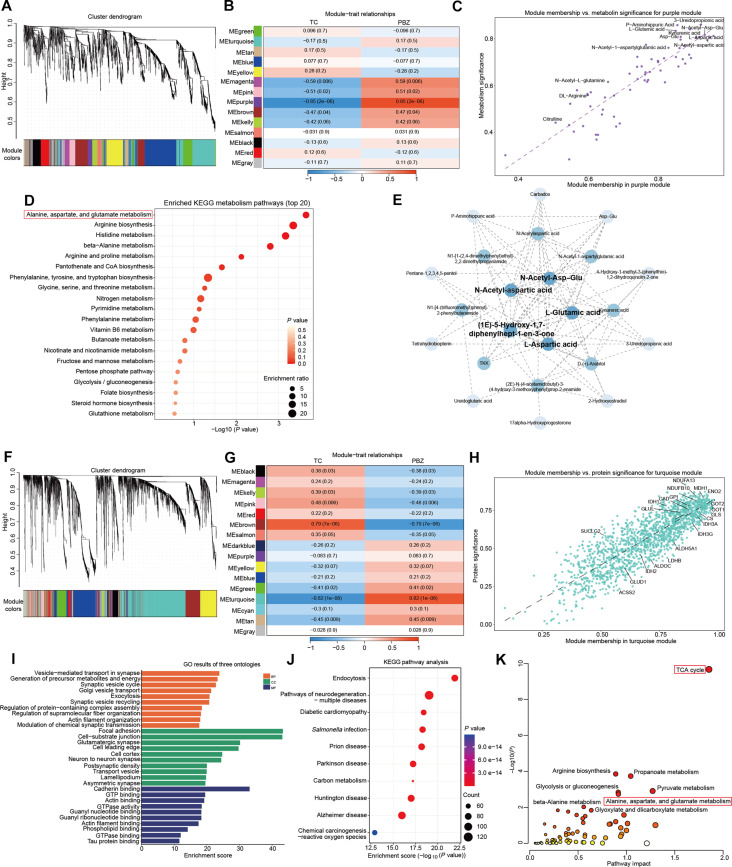
Weighted gene coexpression network analysis (WGCNA) analysis reveals key mechanisms of metabolic reprogramming in glioblastoma. (A) WGCNA clustering dendrogram of GBM tumor core tissues versus peritumoral brain tissues, grouping metabolites into 14 distinct modules (represented by different colors), defined by branch cutting in the dendrogram. (B) Heatmap showing the correlation between different metabolite modules and clinical traits. Positive correlations are indicated in red, negative correlations in blue, with darker colors representing stronger associations. The purple module exhibits the strongest correlation with the tumor core versus peritumoral brain tissue distinction. (C) Scatterplot illustrating the correlation between module eigengene membership and trait significance for metabolites in the purple module of GBM tumor core versus peritumoral brain tissues. (D) Kyoto Encyclopedia of Genes and Genomes (KEGG) functional enrichment analysis of metabolites in the purple module. Dot size represents the enrichment ratio, while color indicates the −log10(*P* value), with all shown pathways exhibiting statistically significant enrichment (*P* < 0.05). (E) Coexpression network visualization of the purple module, where metabolites with topological overlap matrix similarities > 0.18 are connected by dotted lines. (F) WGCNA clustering dendrogram of proteins, grouping them into 16 distinct modules (represented by different colors), defined by branch cutting in the dendrogram. (G) Heatmap depicting the correlation between different protein modules and clinical traits. Positive correlations are shown in red, negative correlations in blue, with darker colors indicating stronger associations. The turquoise module demonstrates the strongest correlation. (H) Scatterplot displaying the correlation between module eigengene membership and trait significance for proteins in the turquoise module. The key enzymes have been marked with a red border. (I) Gene Ontology (GO) enrichment analysis of proteins in the turquoise module. The top 10 enriched pathways are shown for Biological Process (BP), Cellular Component (CC), and Molecular Function (MF), with all depicted pathways exhibiting significant statistical differences (*P* < 0.05). (J) KEGG pathway enrichment analysis of proteins in the turquoise module, highlighting the top 10 statistically significant metabolic pathways. (K) Joint pathway analysis of metabolites in the purple module and proteins in the turquoise module, annotated using KEGG.

As shown in Fig. 5A to C, metabolites were clustered into 14 distinct modules. Among these, the purple module, comprising 63 metabolites, demonstrated the most significant association with GBM. Metabolites including N-acetyl-Asp–Glu, 3-ureidopropanoic acid, L-glutamic acid, L-aspartic acid, and N-acetyl-aspartate exhibited the strongest correlations within this module and showed the highest efficacy in distinguishing TC from PBZ. KEGG enrichment analysis of metabolites within the purple module indicated significant involvement of various amino acid and energy metabolic pathways, with “Alanine, aspartate, and glutamate metabolism” being the most prominently enriched (Fig. 5D). This result is highly consistent with our earlier findings on metabolic abnormalities during the transition from NAT to TC in adult gliomas without subtype differentiation. Subsequently, we constructed a topological overlap matrix (TOM) by filtering metabolites with high topological similarity based on edge weight thresholds. Notably, key amino acids in the “Alanine, aspartate, and glutamate metabolism” pathway—including L-glutamic acid, L-aspartic acid, N-acetyl-Asp–Glu, and N-acetyl-aspartic acid—were consistently identified as hub metabolites (Fig. 5E).

Proteomic WGCNA revealed 16 protein modules (Fig. 5F to H). The turquoise module, containing 1,656 proteins, displayed the most prominent correlation with GBM status. Within this module, key enzymes involved in “Alanine, aspartate, and glutamate metabolism”, such as GLS, GOT1, citrate synthase, GOT2, CAD, GLUL, and ALDH5A1—demonstrated unique and critical roles, suggesting their pivotal functions in GBM progression. Subsequently, pathway enrichment analysis of proteins in the turquoise module further clarified the specific role of these molecules in driving GBM progression (Fig. 5I and J). Joint pathway analysis integrating the key purple metabolite module and the turquoise protein module confirmed that perturbations in the “TCA cycle” and “Alanine, aspartate, and glutamate metabolism” are central features of metabolic reprogramming in GBM, consistent with previous metabolic pathway analysis (Fig. 5K).

### Plasma metabolic landscape in adult glioma patients

To verify the diagnostic abilities of the plasma metabolic biomarkers for glioma, we conducted targeted metabolomic profiling using a 600-MHz NMR quantitative metabolomics platform on 112 plasma samples from glioma patients and healthy controls. A total of 153 plasma metabolites were identified and quantified, encompassing major carbon metabolites, amino acids, and lipids. Twenty QC samples’ overlaid NMR spectra and signal dispersion plots demonstrated stability of the methodology (Fig. S9A and B). Correlation analysis between NMR quantitative results and clinical biochemical measurements confirmed high quantification accuracy (Fig. S9C).

**Fig. 6.**
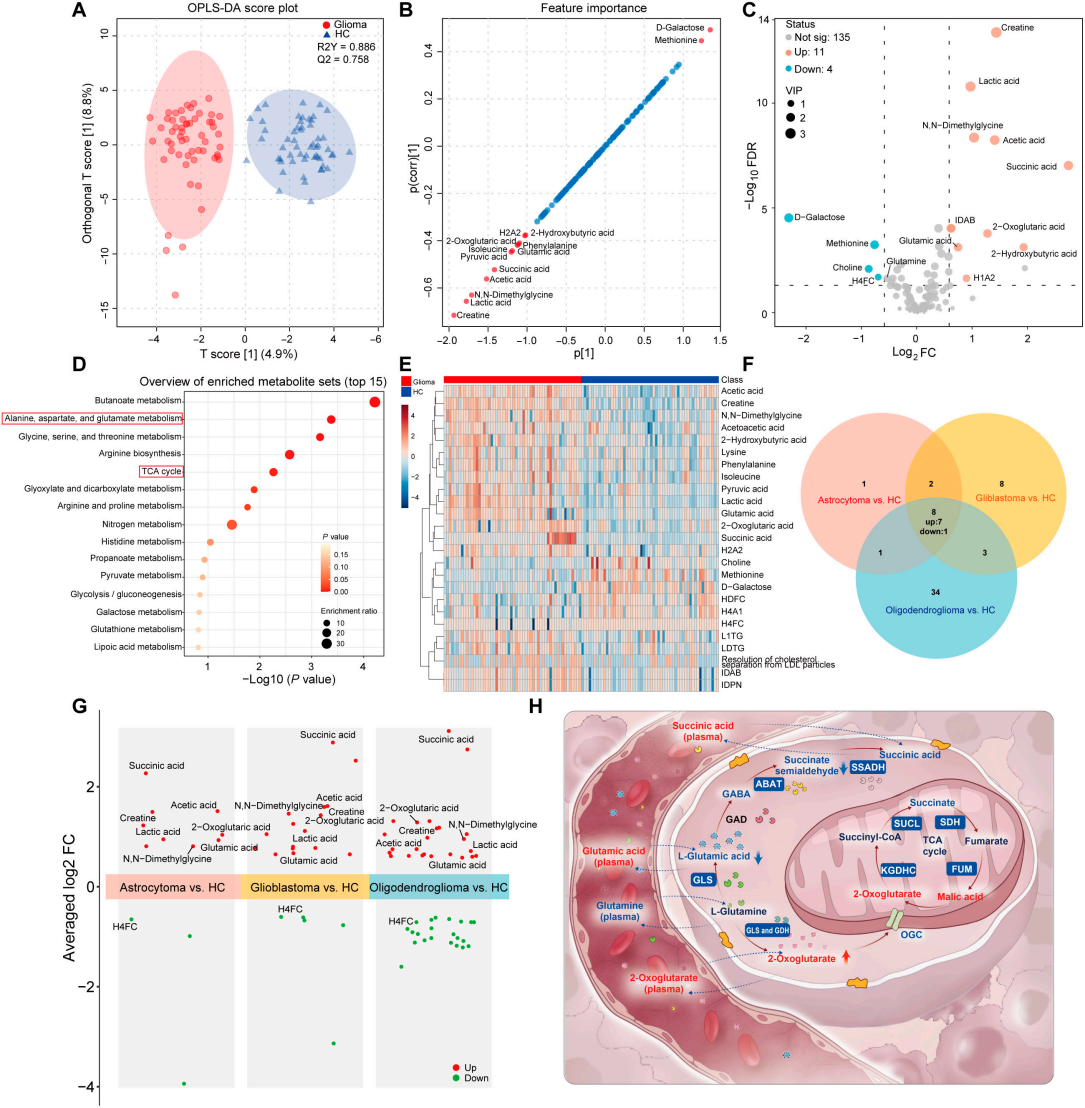
Plasma metabolic landscape in adult glioma patients. (A) Score plot of the orthogonal partial least squares-discriminant analysis (OPLS-DA) model derived from nuclear magnetic resonance-based plasma metabolomics of adult glioma patients (red, *n* = 56) and healthy controls (HC, blue, *n* = 56), with 95% confidence intervals indicated. (B) S-plot of the OPLS-DA model. Metabolites with importance scores greater than 1.0 or less than −1.0 are highlighted in red and labeled, indicating potential biomarkers. Metabolites with importance scores between −1.0 and 1.0 are shown in blue, representing metabolites with lower discriminatory power. (C) Volcano plot displaying differentially abundant plasma metabolites between adult glioma patients and healthy controls, incorporating variable importance in projection (VIP) values. Significance thresholds were set as false discovery rate (FDR) < 0.05 (adjusted using the Benjamini–Hochberg method), fold change (FC) > 1.5 or < 0.667, and VIP > 1.0. Significantly up-regulated and down-regulated metabolites are shown in red and blue, respectively; gray dots denote metabolites with no significant difference. (D) Kyoto Encyclopedia of Genes and Genomes term enrichment analysis of differentially abundant plasma metabolites in adult glioma patients compared to healthy controls. The top 15 significantly enriched metabolism-related pathways are displayed. Dot size represents the enrichment ratio, and color intensity corresponds to the *P* value. (E) Unsupervised hierarchical clustering analysis heatmap of the top 25 plasma metabolites in adult glioma patients and healthy controls. Red indicates relatively higher metabolite abundance; blue indicates relatively lower abundance. (F) Venn diagram illustrating the overlap of significantly differentially abundant plasma metabolites across 3 major adult glioma subtypes. Eight metabolites were commonly dysregulated, with 7 up-regulated and 1 down-regulated. (G) Multigroup volcano plots comparing differential metabolite profiles among major glioma subtypes (glioblastoma, astrocytoma, and oligodendroglioma). Metabolites meeting the significance threshold (*P* < 0.05; FC > 1.5 or < 0.667) are represented by solid circles and labeled; nonsignificant metabolites are omitted. Commonly altered metabolites from Fig. 5H are specifically annotated. (H) Schematic diagram summarizing key metabolic processes in glioma tissue and their correlations with plasma metabolites, highlighting glutamic acid metabolism and the tricarboxylic acid (TCA) cycle. Metabolites and proteins are color coded: red for up-regulation, blue for down-regulation, and black for no significant change. ABAT, 4-aminobutyrate aminotransferase; SSADH, succinate-semialdehyde dehydrogenase; SUCL, succinate-CoA ligase; SDH, succinate dehydrogenase; KGDHC, α-ketoglutarate dehydrogenase complex; FUM, fumarate hydratase; OGC, 2-oxoglutarate carrier; GLS, glutaminase; GAD, glutamate decarboxylase; GDH, glutamate dehydrogenase.

OPLS-DA revealed significant differences in the plasma metabolome between adult glioma patients and healthy controls (Fig. 6A). The reliability of the model was confirmed by external permutation testing (Fig. S9D). An S-plot derived from OPLS-DA highlighted metabolites with the greatest contribution to group separation, annotating those with a model predictive ability correlation (*P* [cov]) > 1.0 or < 1.0 (Fig. 6B). Volcano plot according to the screening criteria (FC > 1.5 or < 0.667, FDR < 0.05, VIP > 1.0) identified 11 significantly up-regulated and 4 significantly down-regulated metabolites in the plasma (Fig. 6C). Notably, 2-oxoglutaric acid was significantly elevated in both the plasma and tumor tissue of glioma patients. On the other side, metabolites increased in patient plasma, including creatine, succinic acid, and glutamic acid, were significantly decreased in the glioma tissue; conversely, methionine was reduced in patient plasma but elevated in the glioma tissue. The expression levels of these potential plasma metabolic markers and their comparative analysis in glioma tissues are detailed in Table 1.

**Table 1.** Statistical data of plasma metabolic biomarkers in adult glioma patients and their expression trends in tumor tissues

Plasma metabolic biomarkers	AUC	Fold change	FDR	Trends in plasma	Trends in tissue
Succinic acid	0.856 (0.776–0.918)	6.68	< 0.001	↑	↓
2-Hydroxybutyric acid	0.725 (0.626–0.822)	3.81	< 0.001	↑	ND
Creatine	0.897 (0.827–0.950)	2.71	< 0.001	↑	↓
Acetic acid	0.837 (0.756–0.902)	2.66	< 0.001	↑	ND
2-Oxoglutaric acid	0.787 (0.694–0.874)	2.42	< 0.001	↑	↑
N,N-Dimethylglycine	0.855 (0.778–0.929)	2.05	< 0.001	↑	ND
Lactic acid	0.874 (0.806–0.923)	1.96	< 0.001	↑	ND
Glutamic acid	0.784 (0.694–0.869)	1.68	< 0.001	↑	↓
Pyruvic acid	0.684 (0.597–0.783)	1.36	0.0039	↑	ND
Phenylalanine	0.699 (0.602–0.784)	1.32	0.0019	↑	↑
Isoleucine	0.726 (0.631–0.814)	1.31	< 0.001	↑	-
Lysine	0.662 (0.572–0.749)	1.25	0.0151	↑	↑
Tyrosine	0.651 (0.541–0.737)	1.20	0.0223	↑	↑
Asparagine	0.609 (0.504–0.708)	1.18	0.137	↑	↓
Citric acid	0.636 (0.533–0.740)	0.87	0.0402	↓	↑
Glutamine	0.748 (0.655–0.838)	0.72	< 0.001	↓	-
Methionine	0.725 (0.622–0.820)	0.59	< 0.001	↓	↑
Choline	0.682 (0.573–0.783)	0.55	0.0043	↓	-
d-Galactose	0.781 (0.688–0.864)	0.20	< 0.001	↓	ND

AUC, area under the curve; ↑, up; ↓, down; -, no significant; ND, not detected; FDR, false discovery rate

Functional enrichment analysis of the 15 differential plasma metabolites indicated that “Butanoate metabolism”, “Alanine, aspartate, and glutamate metabolism”, as well as “TCA cycle” pathways were significantly perturbed (Fig. 6D), demonstrating a general consistency between plasma and tissue metabolic pathway alterations in glioma. Random forest (RF) model (Fig. S9E) identified creatine, lactic acid, N,N-dimethylglycine, acetic acid, succinic acid, d-galactose, glutamic acid, glutamine, and 2-oxoglutaric acid as the most influential metabolites for prediction accuracy (mean decrease in accuracy > 0.010) (Fig. S9F). HCA heatmap illustrated distinct plasma metabolic signatures between glioma patients and healthy controls (Fig. 6E). As shown in Fig. 6F and G, 8 metabolites were commonly dysregulated across 3 major adult glioma pathological subtypes, indicating their potential to reflect shared plasma metabolic features of different glioma subtypes and their promise as biomarkers for liquid biopsy. These findings indicate that there exists a complex and intricate interplay between the metabolic reprogramming features of local glioma tissue and the systemic metabolic shifts in glioma patients (Fig. 6H). Plasma metabolome detection can reflect the metabolic characteristics of glioma, demonstrating underlying clinical application value.

### Diagnostic value of plasma metabolites in glioma patients

To further validate the diagnostic efficacy of plasma biomarkers for glioma liquid biopsy, we established 3 independent external test sets. These sets were used exclusively for performance assessment and were not involved in model training or optimization. The adult glioma test set comprised plasma samples from adult glioma patients diagnosed between March 2023 and August 2025 (*n* = 26) and healthy adult controls (*n* = 26). Given that pediatric gliomas exhibit distinct molecular mechanisms and greater pathological heterogeneity compared to adult gliomas, we also constituted a pediatric test set including pediatric patients with primary brain tumors (*n* = 53, comprising 20 astrocytomas, 8 diffuse midline gliomas, 5 GBM, 2 mixed gliomas, 12 medulloblastomas, and 6 ependymomas) and pediatric nontumor controls (*n* = 53, from patients with intracranial arteriovenous malformations [AVMs]). To further evaluate the tumor specificity, we included an adult pancreatic cancer test set consisting of adult pancreatic cancer patients (*n* = 80) and healthy controls (*n* = 80), considering the metabolic similarities between pancreatic cancer and glioma, particularly the prominent Warburg effect and dysregulated amino acid metabolism [30].

**Fig. 7.**
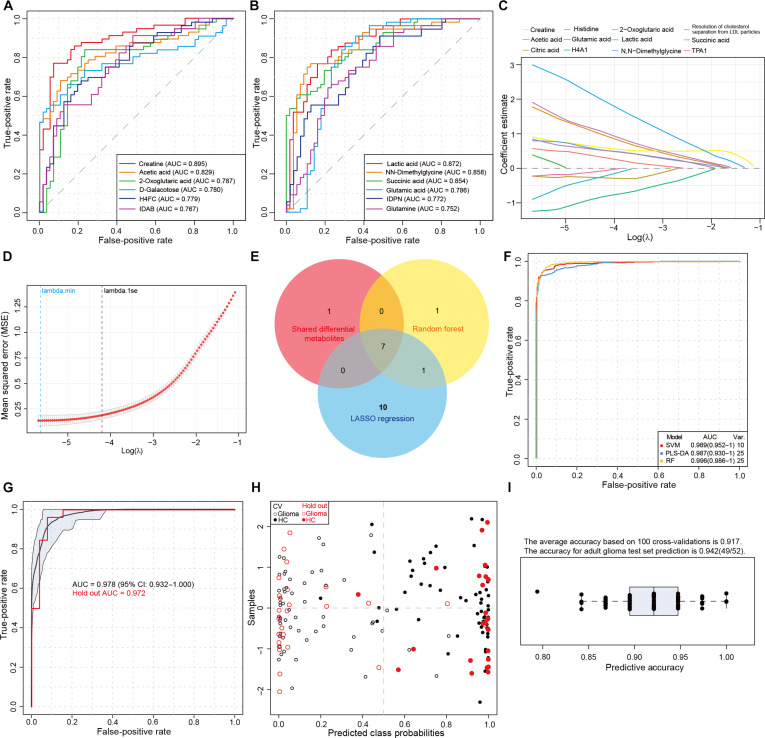
Diagnostic value of plasma metabolites in glioma patients. (A and B) Receiver operating characteristic (ROC) curves of plasma metabolites distinguishing adult glioma patients from healthy controls. 12 metabolites with area under the curve (AUC) values exceeding 0.75 are presented. (C) Characterization of coefficient variation patterns in Least Absolute Shrinkage and Selection Operator (LASSO) regression, revealing the trajectory of coefficient changes across different regularization strengths. (D) Cross-validation plot for tuning parameter λ in LASSO regression. This demonstrates the process of selecting the optimal λ value using 10-fold cross-validation in the LASSO regression model. (E) Venn diagram illustrating the screening strategy for key plasma metabolic markers: (a) differentially abundant in all 3 major glioma subtypes, (b) average importance decrease > 0.010 in random forest (RF) models, and (c) meeting lambda.min criteria in LASSO regression. (F) The ROC curves representing the highest discrimination performance of the support vector machine (SVM) (red), partial least squares-discriminant analysis (PLS-DA) (blue), and RF (yellow) machine learning algorithms, along with the required number of metabolites. (G) ROC curves demonstrating the diagnostic efficacy of a 7-plasma metabolic marker panel validated through cross-validation (based on SVM algorithm). The black curve represents the adult glioma training set using cross-validation, while the red curve shows results from an independent Holdout test set. (H) Scatter plots showing class probabilities for each sample in the SVM-based diagnostic model. Hollow dots represent glioma patients, while solid dots indicate healthy or nontumor control subjects. (I) Prediction accuracy rates of the adult glioma training set based on 100 Monte Carlo cross-validation, along with accuracy rates for the independent Holdout test set validation.

Within the training set, 12 plasma metabolites demonstrated individual diagnostic potential with area under the receiver operating characteristic curve (AUC) values exceeding 0.75 (Fig. 7A and B). The coefficient path plot of the Least Absolute Shrinkage and Selection Operator (LASSO) regression model illustrated the trajectory of coefficients for the top 12 metabolites as the penalty parameter log(lambda, λ) increased (Fig. 7C). Cross-validation (CV) indicated optimal model performance at lambda.min = 0.033 (minimizing deviation) and a parsimonious model at lambda.1se = 0.01 (Fig. 7D). We subsequently constructed diagnostic models using 3 machine learning algorithms: linear support vector machine (SVM), RF, and partial least squares-discriminant analysis (PLS-DA). All 3 models showed comparable and high efficacy (Fig. S10A to C).

To enhance clinical applicability, we prioritized model simplification. Seven metabolites, including creatine, lactic acid, succinic acid, N,N-dimethylglycine, 2-oxoglutaric acid, acetic acid, and glutamic acid, were selected for the model based on stringent criteria: (a) consistent differential expression across 3 adult glioma subtypes; (b) a mean decrease in accuracy > 0.010 in the RF model, and (c) meeting the lambda.min criterion in LASSO regression (Fig. 7E). Algorithm comparison revealed that the SVM algorithm achieved peak diagnostic performance with only 10 features, whereas RF and PLS-DA required 25 features (Fig. 7F and Fig. S10D to F). Consequently, we finalized an SVM-based model integrating these 7 plasma metabolites. This model demonstrated robust performance, with an AUC of 0.978 (95% confidence interval [CI]: 0.932 to 1.000) in 70% CV (Fig. 7G and H) and an AUC of 0.972 in a hold-out test set. Monte Carlo CV repeated 100 times yielded a mean accuracy of 0.917 and a test set accuracy of 0.942 (Fig. 7I). Together, the model based on these 7 key plasma metabolites exhibits high diagnostic accuracy for gliomas, with promising specificity that supports its potential clinical utility for glioma liquid biopsy.

### Development of plasma-based liquid biopsy for glioma

**Fig. 8.**
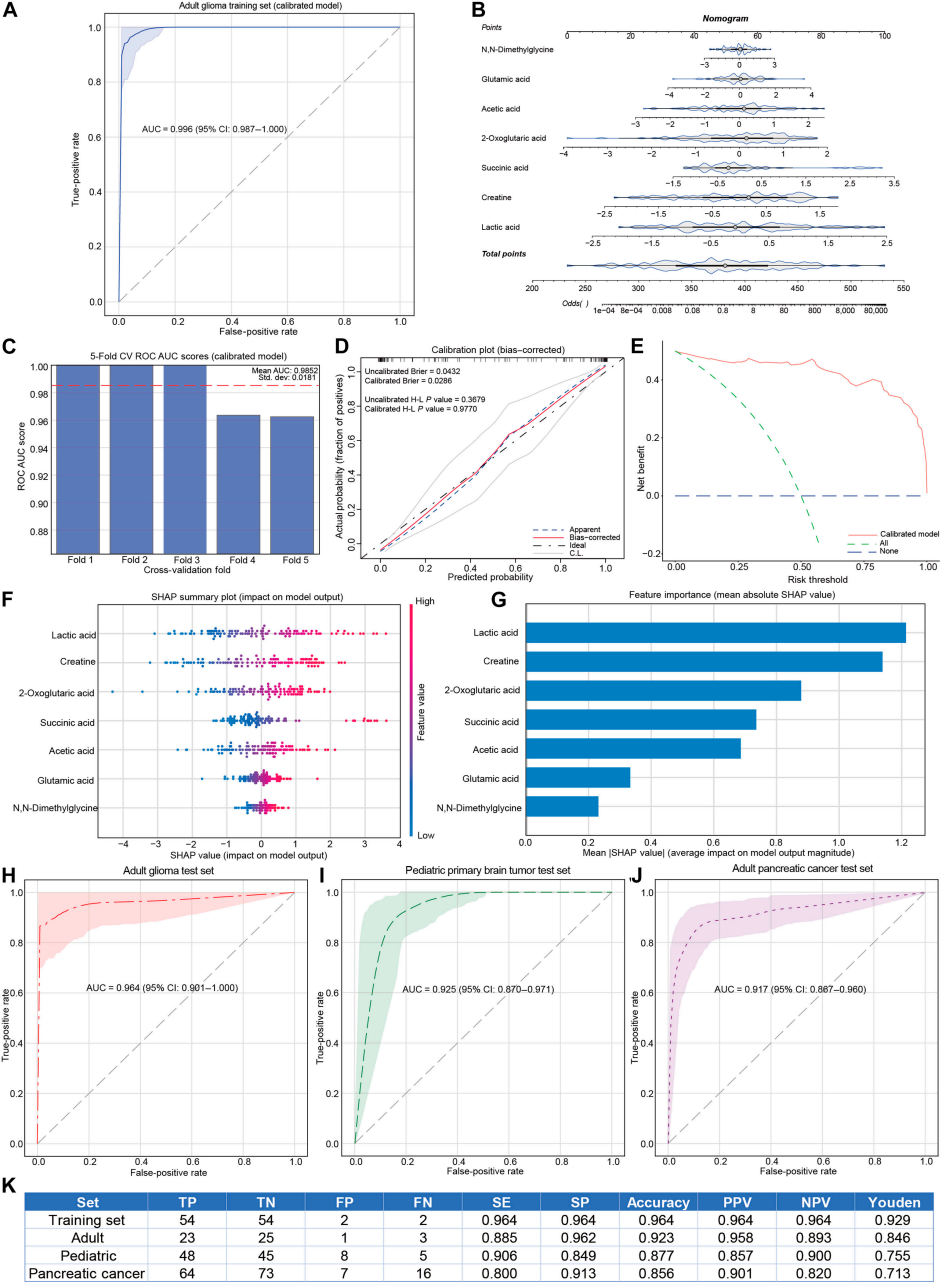
Development of plasma-based liquid biopsy for glioma. (A) Receiver operating characteristic curves based on logistic regression in training set. (B) Nomogram developed using logistic regression. Metabolite quantitative absolute values were log10 transformed and *z*-score normalized. To determine the score associated with a metabolite value, locate the metabolite’s value on the scale, draw a vertical line to the “Points” axis, and sum these individual scores. Then, locate the total score on the “Total Points” axis and draw a vertical line down to the “Risk” axis to obtain the odds ratio for glioma risk. (C) Fivefold cross-validation of the calibrated plasma metabolic biomarker nomogram model for predicting adult glioma. (D) Calibration plot of the plasma metabolic model in training set. (E) Decision curve analysis illustrating the clinical utility of this predictive model in screening for adult glioma. The x-axis represents risk threshold probability, while the y-axis denotes net benefit. The red line indicates the decision curve for plasma metabolic features, and while dashed lines represent 2 reference strategies: “treat-all” (green) and “treat-none” (blue). (F) Shapley additive explanation (SHAP) summary plot explaining the relative importance of individual plasma metabolic biomarkers in the model. (G) Average absolute SHAP values for each plasma metabolic biomarker in SHAP analysis. (H and J) Diagnostic performance of the diagnostic model across 3 independent test sets. (H) Independent adult glioma test set. (I) Independent pediatric primary brain tumor test set. (J) Independent adult pancreatic cancer test set. (K) Receiver operating characteristic information of the training set and 3 independent test sets based on the logistic regression diagnostic model. TP, true positive; TN, true negative; FP, false positive; FN, false negative; SE, sensitivity; SP, specificity; PPV, positive predictive value; NPV, negative predictive value.

To facilitate clinical translation, we constructed diagnostic models using the previously validated model of 7 plasma metabolites via 4 machine learning algorithms: multivariable logistic regression, XGBoost, Gaussian Naive Bayes, and K-Nearest Neighbors. All 4 algorithms yielded models with robust performance, further confirming the efficacy of the plasma metabolomics-based diagnostic approach (Fig. 8A and Fig. S11A to C). Among these, the multivariable logistic regression model demonstrated superior performance, achieving an AUC of 0.996 (95% CI: 0.987 to 1.000).

The multivariable logistic regression model was subsequently transformed into an accessible nomogram for clinical application (Fig. 8B). In the nomogram, each predictor was assigned weighted points based on its regression coefficient, and the total points corresponded to an individual’s odds ratio of having glioma. The model’s robustness was confirmed via 5-fold CV (Fig. 8C). The calibration curve showed excellent agreement between the observed and predicted prevalence of glioma (Fig. 8D). Decision curve analysis (DCA) indicated that the model provided higher net benefit across a wide range of threshold probabilities, verifying its high accuracy and clinical utility (Fig. 8E).

To enhance the interpretability of the model’s predictions and variable interactions, we employed Shapley additive explanation (SHAP) analysis. The SHAP summary plot illustrates the importance and individual contribution of each variable to the nomogram’s predictions (Fig. 8F). Each dot in the plot represents a SHAP value for a specific metabolite in an individual patient, showing how the metabolite’s value (indicated by color) influences the model output. The metabolites are ranked by their mean absolute SHAP value, which reflects their overall contribution to the model. The SHAP mean importance plot indicated that, in descending order of predictive impact, lactate, creatine, 2-oxoglutaric acid, succinic acid, acetate, glutamic acid, and N,N-dimethylglycine were the most significant contributors (Fig. 8G). Furthermore, SHAP dependence further demonstrates the interaction effects between variables and how they influence the model’s predictions (Fig. S11D).

Subsequent external validation showed that the model retained high sensitivity and specificity in independent test sets of both adult and pediatric patients, confirming its robust diagnostic efficacy for glioma (Fig. 8H and I). To investigate whether vascular malformation affects glioma-associated plasma metabolic profiles, we analyzed the plasma metabolomic differences between AVM patients and children with glioma. The results showed that all 7 metabolites in the targeted NMR metabolic panel exhibited significant differences, with trends identical to those in the adult glioma cohort. This confirms that the vascular abnormalities of AVM do not interfere with the glioma-associated plasma metabolic signatures investigated in this study (Table S6). In contrast, when applied to the adult pancreatic cancer test set, the model’s sensitivity decreased significantly (Fig. 8J). Table S7 presents the expression levels and discriminatory efficacy of the 7 in pancreatic cancer. Detailed performance parameters of the model in the training set and the 3 independent test sets are summarized in Fig. 8K.

### Potential of plasma metabolomics-based liquid biopsy for molecular subtyping of glioma

Our study confirmed that the quantitative detection of 7 core metabolites using the targeted NMR platform meets the requirements of accuracy and stability for auxiliary glioma diagnosis. However, the number of metabolites covered by this platform is limited, making it difficult to capture subtle metabolic differences between molecular subtypes. To more comprehensively reveal the metabolomic differences between different molecular subtypes of glioma and provide a richer feature dimension for molecular subtyping, we turned to the LC-MS-based untargeted metabolomic liquid biopsy strategy to further explore the potential of metabolomics-based plasma liquid biopsy in identifying glioma subtypes. A total of 65 plasma samples from glioma patients were included in the LC-MS untargeted metabolomic detection, leading to the identification of 1,459 metabolites. Differential metabolite analysis was conducted based on IDH mutation status and World Health Organization (WHO) grading. Additionally, metabolomic profiles of TC tissues from 110 glioma patients were analyzed to compare tissue-specific metabolic signatures with plasma patterns.

**Fig. 9.**
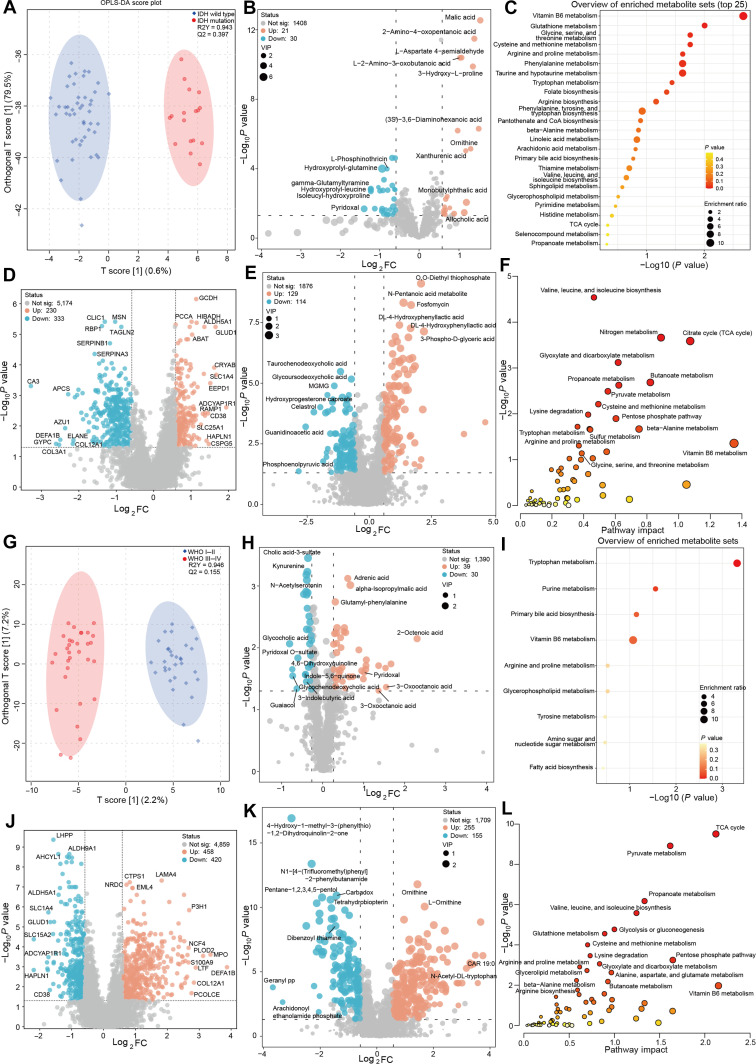
Potential of plasma metabolomics-based liquid biopsy for molecular subtyping of glioma. (A) Orthogonal partial least squares-discriminant analysis (OPLS-DA) score plot comparing isocitrate dehydrogenase (IDH)-mutant (red dots) and IDH-wild-type (blue dots) glioma plasma metabolomes based on liquid chromatography-mass spectrometry, with 95% confidence intervals and model *R*^2^/*Q*^2^ values annotated. (B) Volcano plot displaying differentially abundant plasma metabolites between IDH-mutant and IDH-wild-type gliomas, with variable importance in projection (VIP) values indicated. Significance thresholds: unadjusted *P* < 0.05, fold change (FC) > 1.5 or < 0.667, VIP > 1.0. Up-regulated metabolites are marked in red, down-regulated in blue, and nonsignificant metabolites in gray. (C) Top 25 Kyoto Encyclopedia of Genes and Genomes-enriched metabolic pathways associated with differentially abundant plasma metabolites in IDH-mutant versus IDH-wild-type gliomas. (D) Volcano plot showing differentially expressed proteins in IDH-mutant versus IDH-wild-type glioma tissues. Significance thresholds: unadjusted *P* < 0.05, FC > 1.5 or < 0.667. Up-regulated proteins (*n* = 230) are marked in red, down-regulated (*n* = 333) in blue. (E) Volcano plot of differentially abundant tumor tissue metabolites between IDH-mutant and IDH-wild-type gliomas. Significance thresholds: unadjusted *P* < 0.05, FC > 1.5 or < 0.667, VIP > 1.0. (F) Integrated pathway analysis of metabolomic and proteomic data revealing key metabolic differences between IDH-mutant and IDH-wild-type glioma tissues. (G) OPLS-DA score plot comparing high-grade glioma (red dots) and low-grade glioma (blue dots) plasma metabolomes based on liquid chromatography-mass spectrometry, with 95% confidence intervals and model *R*^2^/*Q*^2^ values annotated. (H) Volcano plot displaying differentially abundant plasma metabolites between high-grade and low-grade gliomas. Significance thresholds: unadjusted *P* < 0.05, FC > 1.2 or < 0.833, VIP > 1.0. (I) Kyoto Encyclopedia of Genes and Genomes-enriched metabolic pathways associated with plasma metabolites differentially abundant between high-grade and low-grade gliomas. (J) Volcano plot of differentially expressed proteins in high-grade versus low-grade glioma tissues. Significance thresholds: unadjusted *P* < 0.05, FC > 1.5 or < 0.667. (K) Volcano plot of differentially abundant tumor tissue metabolites between high-grade and low-grade gliomas. Significance thresholds: unadjusted *P* < 0.05, FC > 1.5 or < 0.667, VIP > 1.0. (L) Joint pathway analysis of metabolomic and proteomic data revealing key metabolic differences between high-grade and low-grade glioma tissues.

As shown in the OPLS-DA loading plot, plasma metabolomes of IDH-mutant and IDH-wild-type glioma patients were significantly separated (Fig. 9A). In IDH-mutant patients, 21 metabolites were significantly up-regulated and 30 down-regulated. Notably, malic acid levels were elevated in the plasma of IDH-mutant patients, whereas pyridoxal was markedly reduced (Fig. 9B). Eleven metabolites achieved AUC values > 0.85, indicating strong discriminatory power for IDH status (Table S8). Functional enrichment analysis revealed that differential plasma metabolites were primarily involved in “Vitamin B6 metabolism”, “Glutathione metabolism”, and “Glycine, serine, and threonine metabolism” (Fig. 9C). Tissue-based proteomic and metabolomic perturbations are visualized in volcano plots (Fig. 9D and E). Joint pathway analysis of tissue data highlighted “Vitamin B6 metabolism” as the top-ranked pathway, consistent with the plasma metabolomic findings (Fig. 9F).

We next assessed the ability of plasma metabolomics to differentiate gliomas by WHO grade, a critical factor for prognosis and therapeutic decision-making. Significant differences were observed between high-grade (WHO III–IV) and low-grade (WHO I–II) glioma (Fig. 9G). In high-grade gliomas, plasma levels of adrenic acid and alpha-isopropylmalic acid were significantly increased, while cholic acid-3-sulfate and kynurenine were decreased (Fig. 9H). Enriched pathways included tryptophan metabolism and purine metabolism (Fig. 9I). In tumor tissues, high-grade gliomas were characterized by more pronounced disruptions in energy metabolism pathways, such as the “TCA cycle” and “Pyruvate metabolism” (Fig. 9J to L). A summary of plasma metabolites with the potential to distinguish high-grade from low-grade gliomas is provided in Table S9.

## Discussion

Glioma cells exhibit a strong infiltrative phenotype, typically invading the PBZ beyond the primary TC [21,31]. Given that PBZ contains functionally critical normal brain tissue, complete surgical resection is frequently challenging, rendering this region a primary site for postoperative recurrence [32]. Thus, efficient and accurate diagnosis holds particular clinical significance for glioma management. In addition to imaging diagnosis and tissue biopsy, liquid biopsy strategies hold great promise as an important component of comprehensive malignant tumor diagnosis. Detectable targets include CTCs, circulating tumor DNA (ctDNA), tumor-derived exosomes, cfDNA methylation markers, and circulating metabolites [14,15,33]. In most cases, these targets can faithfully reflect the specificity and accuracy of tumor tissue origin. The clinical utility of metabolomics-based liquid biopsy has been validated in multiple solid tumors, including esophageal [34], breast [35,36], lung [37,38], and gastric [39] cancers. In glioma, metabolomic-based liquid biopsy confers distinct advantages over larger-size biomarkers such as CTCs or cfDNA, as small-molecule metabolites can readily traverse the fenestrations of the cerebral microvascular endothelium [9]. However, plasma metabolite levels are susceptible to a multitude of confounding factors [40], and whether tissue-specific metabolic signatures can be reliably detected in peripheral plasma remains inadequately investigated [41]. In this study, through an integrated multi-omics approach, we identified metabolic alterations shared between glioma tissue and plasma, enabling the precise identification of glioma-specific plasma biomarkers. On this basis, we developed and optimized a diagnostic model based on 7 plasma metabolites, which exhibits substantial potential for clinical application in glioma liquid biopsy.

Identifying the characteristic metabolic reprogramming events of glioma is a fundamental prerequisite for developing robust metabolomics-based liquid biopsy strategies. To delineate the key metabolic reprogramming events driving glioma progression, we performed LC-MS-based metabolomic and proteomic profiling of glioma tissue specimens, encompassing TC, PBZ, and NAT. In previous studies, PBZ has been defined as the peritumoral edematous region lacking contrast enhancement on T1-weighted gadolinium-enhanced MRI [25]. Histopathological and single-cell sequencing studies have demonstrated that cells within the PBZ display a unique phenotype distinct from both the TC and normal brain parenchyma, representing a transitional intermediate state during the progression from normal tissue to invasive tumor [42,43]. Our findings revealed that “Alanine, aspartate, and glutamate metabolism” and “TCA cycle” pathways are the most significantly perturbed pathways in gliomagenesis and progression. At the metabolite level, L-glutamic acid emerged as a central regulatory node, interacting extensively with molecules involved in amino acid, lipid, nucleotide, and energy metabolisms. Key amino acid metabolites such as L-aspartic acid and L-glutamic acid were significantly down-regulated, whereas intermediates of energy metabolism such as malic acid and 2-oxoglutaric acid were markedly up-regulated. Concurrently, proteomic analysis revealed dysregulation of enzymes implicated in glutamic acid and aspartic acid metabolism, including NAT8L, GLS, GOT1, GLUL, and CAD. Furthermore, the therapeutic potential of targeting these pathways is underscored by ongoing preclinical and clinical investigations into inhibitors of key enzymes such as GLS, GLUD1, and CAD [44–48]. Amino acid metabolism, such as glutamate metabolism, is abnormally active in glioma, and this demand is closely linked to energy metabolic reprogramming such as the Warburg effect [49]. First, glioma cells primarily rely on aerobic glycolysis for energy production, resulting in limited carbon sources entering the TCA cycle. Therefore, amino acids such as glutamine and glutamic acid are extensively converted into intermediates as carbon sources to support the TCA cycle [50]. Second, enhanced biosynthetic activity is a fundamental feature of malignant tumors such as glioma, and amino acids are heavily consumed for the synthesis of lipids and nucleic acids to sustain rapid tumor proliferation [51]. Together, our study confirms that the coordinated down-regulation of amino acid pathway components, alongside the accumulation of energy pathway intermediates, suggests a metabolic rewiring in glioma characterized by glutamate consumption coupled with 2-oxoglutaric acid production. This remodeling likely fuels the rapid proliferation demands by providing both energy and biosynthetic precursors, aligning with the well-recognized Warburg effect and the utilization of alternative carbon sources derived from the tumor microenvironment [52].

Another major challenge in glioma research lies in the high degree of intratumoral and intertumoral heterogeneity. The 2021 WHO classification of central nervous system tumors categorizes gliomas into distinct molecular subgroups based on key genetic alterations, including IDH mutation, epidermal growth factor receptor amplification, 1p/19q codeletion, and cyclin-dependent kinase inhibitor 2A/2B homozygous deletion [53,54]. These distinct driver genetic events underpinning different pathological subtypes give rise to divergent metabolic profiles [16]. A quintessential example is the accumulation of 2-hydroxyglutarate specifically in IDH-mutant gliomas, a metabolic signature that is absent in IDH-wild-type tumors [55]. Several studies have explored metabolomics-based liquid biopsy strategies tailored to specific glioma subtypes, such as adult GBM or pediatric brainstem tumor [56,57]. While these approaches demonstrate favorable diagnostic efficacy for their target populations, their utility is often limited to the specific subtypes they were design for. In the current research, we confirmed that the perturbation in the “Alanine, aspartate, and glutamate metabolism” and “TCA cycle” pathways are conserved across the major glioma subtypes, thereby providing reliable and universal detection targets for the subsequent development of metabolomics-based diagnostic strategies.

There exists a complex interplay between local tumor tissue metabolic reprogramming and systemic metabolic transformation in cancer patients. In pancreatic cancer, tumor-secreted interleukin-1β (IL-1β) can stimulate hepatic gluconeogenesis, leading to systemic hypermetabolism [58]. Breast cancer cells avidly consume glutamine, depleting peripheral blood levels of this amino acid and potentially inducing muscle catabolism [59,60]. These observations raise a critical question: Can plasma metabolomic profiling faithfully reflect the characteristic metabolic shifts occurring within the primary tumor tissue? To address this critical gap, our study integrated metabolomic analyses of both glioma tissue and plasma to investigate their metabolic interplay and identify plasma biomarkers that accurately mirror tumor tissue metabolic characteristics. NMR-based targeted metabolomics revealed that the “Alanine, aspartate, and glutamate metabolism” and the “TCA cycle” pathways were also the most significantly perturbed pathways in the plasma of glioma patients. This finding suggests a high degree of concordance between key metabolic alterations in glioma tissue and the host’s systemic metabolic response. Specifically, metabolites including glutamate, aspartate, creatine, succinic acid, 2-oxoglutaric acid, and methionine were significantly dysregulated in both compartments. Notably, 2-oxoglutaric acid was significantly elevated in both the plasma and tissue of glioma patients. However, an intriguing inverse relationship was observed for several metabolites: Glutamic acid, aspartic acid, succinic acid, and creatine were significantly decreased in glioma tissue but elevated in plasma. This divergent pattern likely reflects a systemic compensatory mechanism in response to the high demand for amino acids and energy by tumor cells. The massive consumption of these metabolites by the tumor may stimulate peripheral organs, such as the liver, to up-regulate their synthesis and accelerate muscle breakdown, thereby reshaping the systemic metabolic landscape and potentially promoting cancer cachexia [22]. Compelling evidence has demonstrated that local metabolic consumption by glioma exerts extensive impacts on host metabolism. Despite the presence of the BBB, glioma tissues still extensively uptake carbon sources from the circulation. A quantitative metabolic flux analysis study by Scott et al. [61] confirmed that glioma extensively scavenges carbon sources such as amino acids from the host’s blood circulation, rewires energy metabolism, and generates a large number of molecules required for proliferation and invasion. This finding has been validated in both animal models and glioma patients, which is highly consistent with the conclusions of our study. Mechanistically, BBB transporters have been shown to play crucial roles in the metabolic crosstalk between glioma and the host. For instance, glutamate transporter-1 has been identified as the major transporter for glioma to uptake glutamate from circulation [62]. Besides, a recent study indicated that the SLC36 amino acid transporter Pathetic (Path) in the BBB is a key molecule regulating the uptake of branched-chain amino acids by glioma from the environment [63]. Down-regulation of Path slows down the cell cycle progression of glial cells, thereby restricting the growth of glioma cells, and up-regulation of Path is sufficient to counteract the inhibitory effect of nutrition restriction on tumor growth. Furthermore, tumor tissues release a variety of metabolism-modulating mediators, including inflammatory cytokines such as IL-6 and tumor necrosis factor-α, as well as regulatory molecules of the systemic metabolic system such as transforming growth factor-β and leptin [22,64]. Tumor-derived exosomes are considered one of the key pathways for glioma to release these mediators [65]. Studies have confirmed that exosomes secreted by glioma can induce normal tissue cells to release a large number of proinflammatory cytokines such as interferon γ and various ILs (such as IL-1A, IL-1B, IL-8, and IL-12), rewiring host metabolism and mediating inflammation [66]. Collectively, these lines of evidence indicate that glioma reshapes host metabolism through multiple pathways to meet the metabolic demands of its rapid growth. This “local tumor consumption-systemic compensation” model provides a novel paradigm for understanding systemic metabolic dysregulation in glioma. It further implies that plasma metabolites can act as indirect sensors of the tumor’s metabolic state, with their dynamic changes holding potential for monitoring tumor progression and therapeutic response. Together, our findings demonstrate that NMR-based metabolomic biomarkers can effectively reflect characteristic molecular events of glioma, establishing this approach as a reliable new strategy for glioma liquid biopsy.

We established a diagnostic model based on a panel of 7 key plasma metabolites. These metabolites exhibited consistent dysregulation across major adult glioma subtypes, accurately reflecting the conserved metabolic characteristics of glioma tissue. Their selection was rigorously validated through dual screening processes employing RF and LASSO regression algorithms, which effectively mitigated potential interference from interindividual heterogeneity. The final model, constructed using a calibrated logistic regression approach, demonstrated outstanding diagnostic performance in an independent external test set for adult glioma, achieving an AUC of 0.964, an accuracy of 0.923, a sensitivity of 0.885, and a specificity of 0.962. This performance is notably superior when compared to cerebrospinal fluid cfDNA, which has a reported detection rate of approximately 80% [7], and plasma ctDNA, which exhibits a mere 14% detection rate in glioma [67]. This finding underscores the advantage of metabolomic approach for distinguishing adult glioma patients from healthy individuals. Although pediatric primary brain tumors differ significantly from adult gliomas in terms of cellular origin and pathogenesis, our model maintained high diagnostic sensitivity in the pediatric primary brain tumors set, achieving an AUC of 0.925. This performance is highly comparable to existing plasma metabolomic models specifically designed for pediatric brainstem tumors (reported AUC = 0.933) [9]. This result suggests that our model successfully captures the core metabolic features fundamental to glioma metabolic reprogramming, rather than relying on age-specific or subtype-incidental variations. Furthermore, the model exhibited commendable tumor-type specificity. Its diagnostic performance in detecting pancreatic cancer was notably attenuated, with the AUC decreasing from 0.964 (for glioma) to 0.917 and sensitivity declining from 0.885 to 0.800. This compels us to further focus on the overlapping effect of plasma metabolic signatures across different tumors. As shown in Table S10, lactic acid, succinic acid, N,N-dimethylglycine, 2-oxoglutaric acid, acetic acid, and glutamic acid were significantly elevated in the plasma of pancreatic cancer patients, enabling effective distinction between pancreatic cancer patients and healthy adults. This result indicates a high similarity in plasma metabolic signatures between pancreatic cancer and glioma patients, particularly in terms of energy metabolism products. This phenomenon has also been well documented in previous studies by others. A study comparing plasma differential metabolites between early-glioma patients and healthy individuals in the Nordic population found that the TCA cycle pathway and the Warburg effect were the most significantly altered pathways in the plasma metabolome of early-glioma patients. Furthermore, lactic acid, 2-oxoglutaric acid, and citrulline were the most prominently changed plasma metabolites in glioma, showing high consistency with our study on the Asian population [18]. Another study revealed that several plasma metabolites, including 2-oxoglutaric acid, arginine, aspartic acid, glutamic acid, choline, and N,N-dimethylglycine, also exhibited significant changes in early esophageal cancer patients. These findings suggest that these common plasma metabolites may be associated with multiple malignant tumors [34]. One possible explanation for the presence of these common biomarkers across various malignant tumors is that they reflect shared conditions such as metabolic reprogramming and systemic inflammatory responses among tumors. These lines of evidence indicate that the overlapping effect of plasma metabolic signatures may be one of the major challenges limiting the tumor specificity of such diagnostic schemes. Nevertheless, creatine were significantly increased in the plasma of glioma patients, while showing no statistical significance in pancreatic cancer. This suggests that their plasma metabolic signatures are not entirely identical, laying a theoretical foundation for plasma metabolomics-based liquid biopsy to distinguish malignant tumors with highly similar metabolic features.

The NMR-based plasma metabolomics platform demonstrates excellent analytical stability and enables precise quantification of metabolites without requiring expensive isotopic internal standards. This translates to lower per-sample costs compared to alternative liquid biopsy technologies such as mass spectrometry (MS) or ctDNA analysis, making it particularly suitable for large-scale clinical screening applications. Successful clinical translation of high-throughput NMR metabolomics has already been achieved in large epidemiological studies and biobanking initiatives in several countries, including the United Kingdom and Canada [68–70]. Our findings further corroborate that the quantitative accuracy of NMR for metabolite detection is highly comparable to that of standard clinical biochemical assays, establishing it as a reliable and user-friendly platform for metabolomic analyses. From a clinical translation perspective, the logistic regression model developed in this study was transformed into an accessible nomogram, providing a visual tool for risk calculation. DCA indicated that the model provides a superior net benefit across a wide range of threshold probabilities compared to alternative strategies. This feature is crucial for practical implementation, as it allows for rapid estimation of an individual’s glioma risk using the nomogram alone, without the need for complex computational infrastructure. This simplicity makes the model particularly advantageous for use in primary care settings or large-scale screening programs, especially in resource-limited environments. It is acknowledged that current and foreseeable future diagnosis of presurgical glioma continues to rely heavily on contrast-enhanced MRI and other advanced imaging techniques. However, the scarcity of both high-end imaging equipment and expert neuroradiologists, particularly in low-income countries, represents a considerable real-world constraint. It is estimated that comprehensive diagnostic imaging systems are accessible in fewer than 30% of countries and regions globally, leading to substantial delays in glioma diagnosis for a vast number of patients [9]. This reality underscores the value of our research: A liquid biopsy strategy capable of effectively identifying glioma across different age groups and molecular subtypes holds considerable potential to improve diagnostic accessibility. It could serve as a valuable complementary or triage tool, helping to address critical gaps in global neuro-oncologic care.

Nevertheless, this study has several limitations. First, although our research included hundreds of patients and a comparable number of healthy controls, all study cohorts consisted exclusively of individuals of Chinese descent, resulting in relatively homogeneous clinical and metabolic characteristics. The generalizability of our findings to other ethnic populations requires further validation in multi-ethnic cohorts. Second, given the considerable heterogeneity among glioma subtypes, the sample sizes for certain subgroups were relatively limited when stratified by age and specific pathological types. This constraint may affect the robustness of subtype-specific analyses. To address this, we have initiated the development of a plasma-based diagnostic framework for glioma and plan to evaluate the universality of this strategy in larger, geographically diverse, multicenter cohorts. Third, while our study delineated specific metabolites, proteins, and significantly perturbed metabolic pathways potentially critical to glioma pathogenesis and progression, and proposed or substantiated promising therapeutic strategies, further fundamental biological validation is imperative to confirm the therapeutic relevance of these metabolic regulatory networks. For instance, although we identified alterations in metabolites and enzymes that have been linked to prognosis in glioma, their precise roles and the efficacy of targeting them necessitate deeper mechanistic investigation and experimental validation. Notwithstanding these limitations, our findings robustly indicate that plasma metabolic biomarkers can accurately reflect the metabolic reprogramming characteristics of glioma tissue, underscoring their high potential for clinical translation. The development of accessible diagnostic models, such as nomograms based on key metabolites, holds promise for improving diagnostic accessibility, particularly in resource-limited settings where advanced imaging might be scarce.

## Conclusion

In conclusion, our study demonstrates that dysregulations in “Alanine, aspartate, and glutamate metabolism” and “TCA cycle” represent key metabolic reprogramming features during glioma progression. Plasma metabolomic profiling can faithfully reflect the metabolomic characteristics of gliomas. The NMR-based metabolomic clinical diagnostic model exhibits high accuracy and specificity in glioma detection regardless of patients’ age and pathological features, thereby serving as a potential noninvasive tool for glioma screening or auxiliary diagnosis.

## Methods

### Study population and participant recruitment

From January 2022 to August 2025, a total of 566 participants were recruited at Zhujiang Hospital, Southern Medical University. The cohort included 351 treatment-naïve patients clinically diagnosed with glioma or pancreatic cancer and 215 nontumor control participants. All tumor patients received a preoperative clinical diagnosis based on contrast-enhanced MRI or other appropriate imaging modalities. Definitive diagnosis was confirmed postoperatively by histopathological examination of surgical resection specimens according to the WHO classification criteria.

Among the 264 patients initially diagnosed with glioma, 34 were excluded based on the following criteria: (a) a history of other concurrent malignant tumors; (b) final postoperative pathological diagnosis of germ cell tumors, metastatic tumors, or benign lesions; (c) improper handling or preservation of tissue or plasma specimens. Consequently, 177 adult glioma patients and 53 pediatric patients with primary intracranial tumors were enrolled in the study. Tissue and plasma sample availability varied among the adult glioma cohort: 95 patients provided tumor tissue samples only, 55 provided plasma samples only, and 27 provided matched pairs of both plasma and tumor tissue. From the 82 adult glioma patients with available plasma samples, the first 56 consecutive patients enrolled before March 2023 were assigned to the training set, while the subsequent 26 patients formed the temporal-independent test set. All 53 pediatric patients with primary intracranial tumors provided plasma samples only. Among the 87 patients with a preoperative diagnosis of pancreatic cancer, 7 were excluded after postoperative pathology confirmed benign pancreatic lesions, resulting in 80 pancreatic cancer patients included in the final analysis. The control group consisted of 215 nontumor participants. The 162 adult controls were recruited from a healthy population without diagnosed tumors or metabolic diseases. Considering clinical feasibility and ethical issues, children with nontumor neurological diseases were enrolled as the nontumor control group. The 53 pediatric controls were selected from patients hospitalized in the Department of Neurosurgery at Zhujiang Hospital for intracranial AVM. These pediatric controls were screened to exclude individuals with tumors, metabolic diseases, or relevant medical histories during hospitalization. All case and control groups were matched for sex and age.

Sample size estimation was conducted based on established methodologies for multi-omics studies. A previous study utilizing the MultiPower computational framework for multi-omics power analysis recommended a minimum of 16 samples per group for metabolomics and other omics analyses [71]. The sample size for each subgroup in our study met or exceeded this threshold. For the development of the clinical diagnostic model, the sample size was calculated using the events-per-variable criterion. Based on prior research experience, the expected number of predictor variables typically ranges from 5 to 8, requiring approximately 50 to 80 glioma patients. Our study ultimately included 82 diagnostically confirmed adult glioma patients and 53 pediatric patients for the development of the clinical diagnostic model, along with an equal number of matched nontumor controls, thus meeting the calculated sample size requirements for robust model development.

### Sample collection and LC-MS metabolomic analysis

LC-MS analysis was performed on all glioma tissue samples and a subset of glioma plasma samples using a Q Exactive HF-X mass spectrometer (Thermo Fisher Scientific, USA) coupled to a Vanquish ultrahigh performance liquid chromatography system (Thermo Fisher Scientific) [72,73]. Chromatographic separation was achieved using an ACQUITY UPLC BEH Amide column (2.1 mm × 100 mm, 1.7 μm; Waters, Ireland) maintained at 25 °C. High-purity reagents were used throughout the study: Ammonium acetate (NH4AC) and ammonium hydroxide (NH4OH) were purchased from Sigma-Aldrich; acetonitrile (ACN) and methanol were obtained from Merck and Fisher Scientific, respectively.

Peripheral blood samples (1 to 2 ml) were collected from fasting participants via the median cubital vein into 5-ml Vacutainer tubes containing EDTA as an anticoagulant. The samples were centrifuged at 1,500 × g for 15 min at 4 °C to separate plasma from cellular components. A 150-μl aliquot of the supernatant plasma was aliquoted and stored at −80 °C until subsequent analysis. For metabolite extraction, frozen plasma samples were thawed on ice. A 100-μl aliquot was mixed with 400 μl of cold methanol/ACN (1:1, v/v) to precipitate proteins. The mixture was vortexed, incubated on ice for 10 min, and then centrifuged at 14,000 × g for 20 min at 4 °C. The resulting supernatant was collected and completely dried using a vacuum centrifuge. For LC-MS analysis, the dried metabolites were reconstituted in 100 μl of ACN/water (1:1, v/v), followed by centrifugation at 14,000 × g for 15 min at 4 °C. The final supernatant was transferred to an LC-MS vial for injection.

Fresh glioma tissue samples were obtained during surgery by attending neurosurgeons, with the tumor location confirmed using preoperative MRI and intraoperative navigation systems. Tissue specimens were acquired via radical resection or extended resection and immediately flash frozen in liquid nitrogen to quench metabolic activity. Approximately 80 mg of frozen tissue was sectioned on dry ice and transferred to a 2-ml Eppendorf tube. The tissue was homogenized with 200 μl of ultrapure water and 5 ceramic beads using a precooled homogenizer. Subsequently, 800 μl of cold methanol/ACN (1:1, v/v) was added to extract metabolites. The homogenate was subjected to the same centrifugation, drying, and reconstitution steps as described for plasma samples.

Chromatographic separation was performed using hydrophilic interaction liquid chromatography. The mobile phase consisted of (A) 25 mM ammonium acetate and 25 mM ammonium hydroxide in water, and (B) ACN. The gradient elution program was as follows: 98% B held for 1.5 min, linearly decreased to 2% B over 10.5 min, maintained at 2% B for 2 min, rapidly increased to 98% B in 0.1 min, and re-equilibrated for 3 min. The flow rate was 0.3 ml/min, and the injection volume was 5 μl. Mass spectrometric detection was conducted in both positive and negative electrospray ionization modes. The ion source parameters were set as follows: ion source gas 1 (Gas1) as 60, ion source gas 2 (Gas2) as 60, curtain gas (CUR) = 30 psi, source temperature as 600 °C, and ion spray voltage floating as ± 5,500 V. For full-scan MS acquisition, the mass range was set to 80 to 1,200 Da, with a resolution of 60,000 and an accumulation time of 100 ms. For data-dependent tandem MS acquisition, the instrument was set to acquire over the range 70 to 1,200 Da, and the resolution was set to 30,000, with an accumulation time of 50 ms and a dynamic exclusion time of 4 s.

Raw LC-MS data files were converted from the proprietary format to mzXML files using ProteoWizard MSConvert. Preprocessing, including peak picking, alignment, and retention time correction, was performed using the XCMS package in R. For peak picking, the following parameters were used: centWave m/z = 10 ppm, peakwidth = c (10, 60), prefilter = c (10, 100). For peak grouping, bw = 5, mzwid = 0.025, minfrac = 0.5 were used. Annotation of isotopes and adducts was carried out using the CAMERA (Collection of Algorithms for METabolite pRofile Annotation) package. Metabolic features were retained only if they exhibited nonzero measurements in more than 50% of the samples within at least 1 experimental group. Metabolite identification was achieved by matching the accurate mass (mass error < 10 ppm), retention time, and tandem MS fragmentation patterns against an in-house standard database [74,75]. The confidence level of identification was consistent with Level 2 (putative annotation) or higher, as defined by the Metabolomics Standards Initiative [27,28].

### LC-MS proteomic analysis

Protein extraction and proteomic analysis were performed on all tumor tissue samples using liquid chromatography coupled to a Q Exactive HF mass spectrometer (Thermo Fisher Scientific). Frozen tissue samples were retrieved from −80 °C storage and pulverized into a fine powder using a mortar and pestle under liquid nitrogen. The powdered tissue was rapidly transferred to a precooled centrifuge tube and homogenized in an appropriate volume of 4% sodium dodecyl sulfate lysis buffer. The homogenate was vortexed, briefly centrifuged, and subjected to ice-water bath ultrasonication for 8 min to ensure complete lysis. Following low-temperature centrifugation, the supernatant was carefully transferred to a new tube for subsequent processing. An aliquot of 5 μl of the supernatant was reserved for protein quantification, while the remainder was used for reduction and alkylation. Specifically, the main sample aliquot was treated with dithiothreitol solution and incubated at 56 °C for 30 min in a dry bath shaker to reduce disulfide bonds. Subsequently, iodoacetamide solution was added for alkylation, which proceeded in the dark for 30 min. Protein concentration was determined using the bicinchoninic acid (BCA) assay. The reserved 5-μl aliquot was diluted with 15 μl of lysis buffer. A bovine serum albumin standard curve (0.5 μg/μl, with volume gradients from 0 to 20 μl) was prepared alongside the diluted samples in a 96-well plate. Both standards and samples were brought to a final volume of 20 μl with lysis buffer or phosphate-buffered saline, respectively. Sample concentrations were measured in triplicate. A background control containing 20 μl of lysis buffer was included. Then, 200 μl of BCA working reagent (prepared at a 50:1 ratio of BCA solution to Cu reagent) was added to each well, and the plate was incubated at 37 °C for 10 to 20 min. The absorbance was measured at 595 nm using a microplate reader, and sample protein concentrations were calculated based on the standard curve.

Based on the quantified protein concentrations, an aliquot containing 60 μg of protein from each sample was aliquoted for tryptic digestion. The sample was processed using a SP3 (Single-Pot, Solid-Phase-enhanced Sample Preparation) protocol for protein cleanup and digestion. Briefly, 10 μl of magnetic beads were added to the protein aliquot along with a specific volume of 1% formic acid and ACN to a final concentration of 50%. The mixture was incubated on a vortex mixer for 8 min. The beads were then washed sequentially with 70% ethanol and 100% ACN for purification. Following cleanup, the beads were resuspended in 20 μl of digestion buffer containing trypsin and ammonium bicarbonate buffer, and digestion was carried out at 37 °C for over 3 h. A second aliquot of 1 μl of trypsin was added for a further 3-h incubation to ensure complete digestion. After digestion, peptides were purified by adding ACN to a final concentration of 95%, followed by washing steps with ACN. Finally, peptides were eluted from the beads using 2% ACN, aided by ultrasonic oscillation for 30 s. The beads were separated on a magnetic rack, and the supernatant containing the purified peptides was collected. For LC-MS analysis, an amount equivalent to 8 μg of the original protein sample was mixed with the iRT Kit (Biognosys) for retention time calibration. The mixture was centrifuged at 20,000 × g for 10 min, and the supernatant was transferred to an LC-MS vial for injection. Approximately 6 μg of peptides was loaded per analysis.

Data were acquired in a DIA mode to achieve comprehensive peptide profiling. The raw data files generated from the MS analysis were processed using the DIA-NN software suite for database searching and protein identification. The analysis included QC assessments of the MS data, such as peptide and protein mass tolerance distribution analysis and precursor ion mass error distribution, to ensure data reliability. Protein identification was performed by matching the experimentally obtained mass spectra against a specified protein sequence database.

### NMR-based plasma metabolomic profiling and quantification

Plasma samples from all enrolled glioma patients and nontumor participants were subjected to targeted metabolomic profiling using a 600-MHz NMR spectrometer (Bruker Biospin AG). The plasma collection protocol followed the same procedure as described in Section “Sample collection and LC-MS metabolomic analysis”. Briefly, upon thawing, 340 μl of plasma was mixed with an equal volume of NMR-specific plasma buffer (Bruker Plasma Buffer) in a 1:1 ratio. The mixture was vortexed thoroughly, and 600 μl of the solution was transferred into a 5-mm NMR tube (Bruker NMR Tubes) for analysis. All samples were arranged sequentially in an automated sample rack and loaded into the sample handler.

Prior to sample analysis, a QC standard was used to perform a preliminary instrument self-check, ensuring optimal instrument performance, stable environmental temperature, and satisfactory quantitative capability. Samples were introduced into the magnet via an automatic sample handler maintained at 280 K to preserve sample integrity. Before insertion, each sample was preheated using a gas flow system to reach the target detection temperature of 310 K. 1H NMR spectra were acquired under standardized temperature control conditions. The raw spectra were processed using the QuantRef management system integrated into Bruker Topspin software. Spectral normalization was performed to express signal intensities in millimolar (mmol/l) proton concentration equivalents. Chemical shift alignment was calibrated using the trimethylsilylpropanoic acid reference signal and the doublet of alanine at 1.48 ppm. Metabolite identification was conducted by matching the observed spectral patterns against the Bruker in-house NMR metabolite library. For quantitative analysis, metabolite concentrations were determined by integrating signal regions corresponding to characteristic functional groups: the –CH_3_ group at 0.8 ppm and the –CH_2_– group at 1.25 ppm. This approach enabled the absolute quantification of metabolites based on their proton signatures.

In accordance with the Clinical and Laboratory Standards Institute guidelines, the analytical robustness and repeatability of the NMR platform were rigorously evaluated. A pooled QC sample was prepared by combining 0.5 ml of EDTA-plasma from 20 patients, which was then aliquoted into 20 equal portions and stored at −80 °C. Throughout the validation study, 1 aliquot was retrieved and processed twice daily (with an 8-h interval between measurements) over a 10-d period, simulating interday and inter-run variability. The resulting NMR data from the 20 repeated measurements were analyzed using MedCalc software (version 15.2.2). The stability of the metabolomic quantification was assessed by calculating the coefficient of variation (%) for 6 key plasma metabolic indicators: triglycerides, total cholesterol, low-density lipoprotein cholesterol, high-density lipoprotein cholesterol, apolipoprotein A1, and apolipoprotein B. The interassay coefficient of variations for these metabolites ranged between 1.39% and 2.62% (Fig. S9C). Moreover, the levels of key metabolites detected by NMR in healthy control participants all fell within the clinical reference ranges (Table S10), confirming high repeatability and operational stability of the NMR platform under the established analytical conditions.

### Statistical analysis

Statistical analysis, and visualization for multi-omics analysis in this study were performed using SIMCA software (version 14.1; Umetrics, Sweden), MetaboAnalyst 6.0 (https://www.metaboanalyst.ca), and R software environment (version 4.3.3).

Prior to statistical analysis, raw data values were subjected to a standardized preprocessing pipeline. Log10 transformation was applied to all data to approximate a normal distribution, followed by *z*-score normalization to standardize the scale of each variable. To minimize artifacts from low-abundance signals, metabolites or proteins with more than 20% missing values were excluded from subsequent analyses.

For univariate analysis, differences between groups were assessed based on data distribution. For normally distributed data, Student *t* test (unpaired data) or paired *t* test (paired data) was used. For non-normally distributed data, the nonparametric Mann–Whitney U test (unpaired data) or Wilcoxon signed-rank test (paired data) was applied. The FDR was controlled using the Benjamini–Hochberg procedure, and an adjusted *P* < 0.05 was considered statistically significant. These analyses were conducted within the MetaboAnalyst 6.0 platform and R environment.

Multivariate analysis included PCA for unsupervised exploration of data clustering, and OPLS-DA for supervised modeling to maximize group separation. The OPLS-DA models were mean centered and scaled to unit variance. Model robustness was evaluated using a 1,000-permutation test and cross-validated analysis of variance (ANOVA) (CV-ANOVA). Models with a CV-ANOVA *P* ≥ 0.05 or a predictive goodness of fit (*Q*^2^) < 0.1 were considered nonsignificant and rejected. Volcano plots were used to visualize the significance (−log10 *P* value or FDR) versus magnitude of change (log2 FC) of each metabolite or protein. Unsupervised HCA heatmaps were generated to visualize patterns in metabolite profiles across samples. To investigate the relationship between the metabolome and proteome in glioma tissue, O2PLS-DA and the Mantel test were employed to assess correlation and identify covarying features between the 2 omics layers. Debiased sparse partial correlation analysis and metabolite–protein interaction networks were constructed to identify key nodal molecules. WGCNA was used to identify coexpression modules of metabolites and proteins associated with GBM. Functional interpretation of differentially expressed metabolites and proteins was performed using pathway enrichment analysis. KEGG pathway analysis was used for metabolites. For proteins, GO terms, KEGG pathways, and GSEA terms were conducted. Joint pathway analysis was subsequently applied to identify biological pathways commonly enriched by both differential metabolites and proteins.

The diagnostic performance of individual metabolites and metabolite panels was evaluated using receiver operating characteristic curves. Machine learning algorithms, including SVM, PLS-DA, and RF, were used to build and optimize classification models. A Monte Carlo CV with balanced subsampling was implemented: In each iteration, two-thirds of the samples were used for feature selection and model training, while the remaining one-third was used for validation. This process was repeated 100 times to calculate the model’s performance metrics (such as AUC, sensitivity, specificity, accuracy, and Youden’s index) and their 95% CIs. The optimal number of features was determined by maximizing the accuracy. A final multivariate logistic regression model was developed and presented as a nomogram. The nomogram’s calibration was assessed using a calibration curve and the Hosmer–Lemeshow test. Its clinical utility was evaluated using DCA curve. To interpret the contribution of each variable in the model, SHAP analysis was performed. These advanced analyses were carried out using relevant packages in the R software environment.

## Ethics Approval

The study protocol was rigorously reviewed and approved by the Medical Ethics Committee of Zhujiang Hospital, Southern Medical University (2023-KY-022-02). All procedures involving human participants adhered to the ethical principles outlined in the Declaration of Helsinki and complied with relevant national and institutional guidelines for biomedical research. Prior to sample collection, written informed consent was obtained from all participating patients or their legal guardians.

## Data Availability

The datasets of demographic, clinical, and pathological data supporting the conclusions of this article are provided in the Supplementary Materials. The original datasets can be obtained from the corresponding author, H.G., upon reasonable request via email: guohongbo911@126.com.
