# Supplementary material for: Integrative Multi-Omics Analysis Reveals the Characteristic Metabolic Signature of Glioma and Enables Plasma-Based Liquid Biopsy
Source: Research (Wash D C). 2026 Mar 23;9:1199. doi: 10.34133/research.1199 (PMC13006737; doi:10.34133/research.1199)
Supplement: Supplementary 1 — Figs. S1 to S11 Tables S1 to S10 Data files S1 and S2 [file research.1199.f1.zip › Supplementary Materials.docx]

**Supplementary Materials**

Integrative Multi-Omics Analysis Reveals the Characteristic Metabolic Signature of Glioma and Enables Plasma-Based Liquid Biopsy

Yixiao Jiang^1^†, Yufei Lan^1^*†, Yifeng Wang^1^†, Sui Chen^1^, Yixiong Shen^1^, Shiyao Chu^1^, Yaoyuan Dong^1^, Lei Li^1^, Huan Zhang^1^, Zhijie Lu^1^, Yuankai Wang^1^, Jiankun Lu^1^, Xiaoman Li^1^, Feiyunduo Hao^1^, Qu Yue^1,2^*, and Hongbo Guo^1^*

*^1^Neurosurgery Center, The National Key Clinical Specialty, The Engineering Research Center of Diagnostic and Therapeutic Technology and Devices for Cerebrovascular Diseases in Ministry of Education, Guangdong Provincial Key Laboratory on Brain Function Repair and Regeneration, Zhujiang Hospital Institute for Brain Science and Intelligence, Zhujiang Hospital, Southern Medical University, Guangzhou, 510282, China.*

*^2^Department of Functional Neurosurgery, Zhujiang Hospital, Southern Medical University, Guangzhou, 510282, China.*

*Address correspondence to: [guohongbo911@126.com](mailto:guohongbo911@126.com) (H.G.); qu.yue@foxmail.com (Q.Y.); lanyufei686@163.com (Y.L.)

†These authors contributed equally: Yixiao Jiang, Yufei Lan, Yifeng Wang.

**Table of contents**

Figures S1 to S11

Tables S1 to S10

**Supplementary Figures**

**
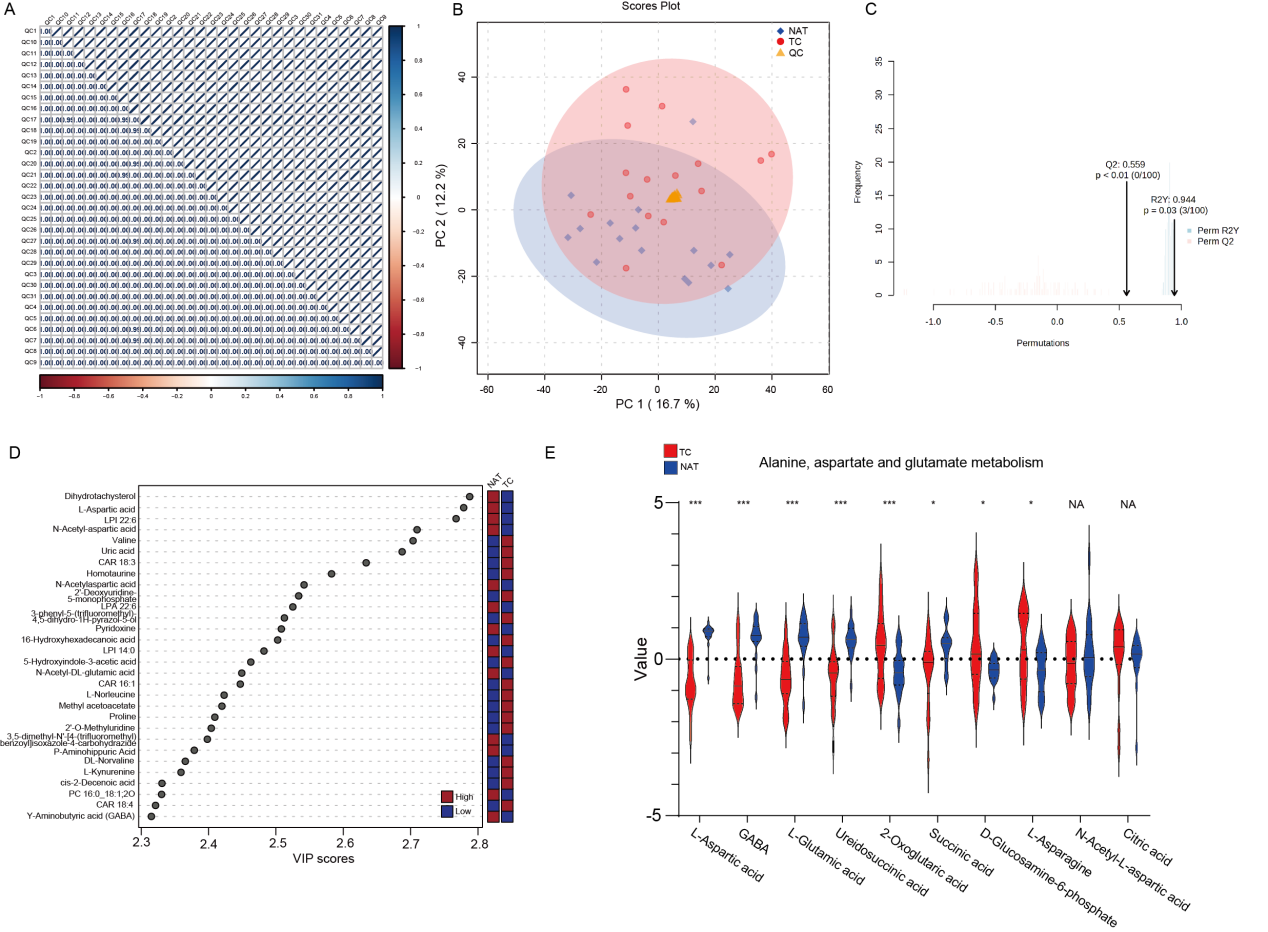
**

**Fig. S1.** Metabolomic landscape of glioma tissues based on LC-MS. (A) Correlation matrix of quality control (QC) samples based on Pearson correlation coefficient analysis, indicating that the correlation coefficients among QC samples are all above 0.9, suggesting good experimental reproducibility. (B) PCA score plot displaying the distribution of samples from different groups. Blue: adjacent normal brain tissues; red: tumor cores; yellow: quality control samples. (C) Permutation test of the OPLS-DA model performed 100 times. R² measures the goodness of fit, while Q² assesses the predictive ability of the model. (D) Metabolites based on VIP values derived from the OPLS-DA model, used to measure the contribution of each metabolite to group separation. The top 30 metabolites with the highest VIP values are shown in the plot. (E) Accordion plots showing the relative abundances of metabolites involved in "Alanine, Aspartate, and Glutamate Metabolism" in tumor cores compared to adjacent normal brain tissues. Red represents tumor cores, and blue represents adjacent normal brain tissues. P-values were determined by paired t-tests, with *** indicating *P* < 0.001, * indicating *P* < 0.05, and NA indicating no significant difference.


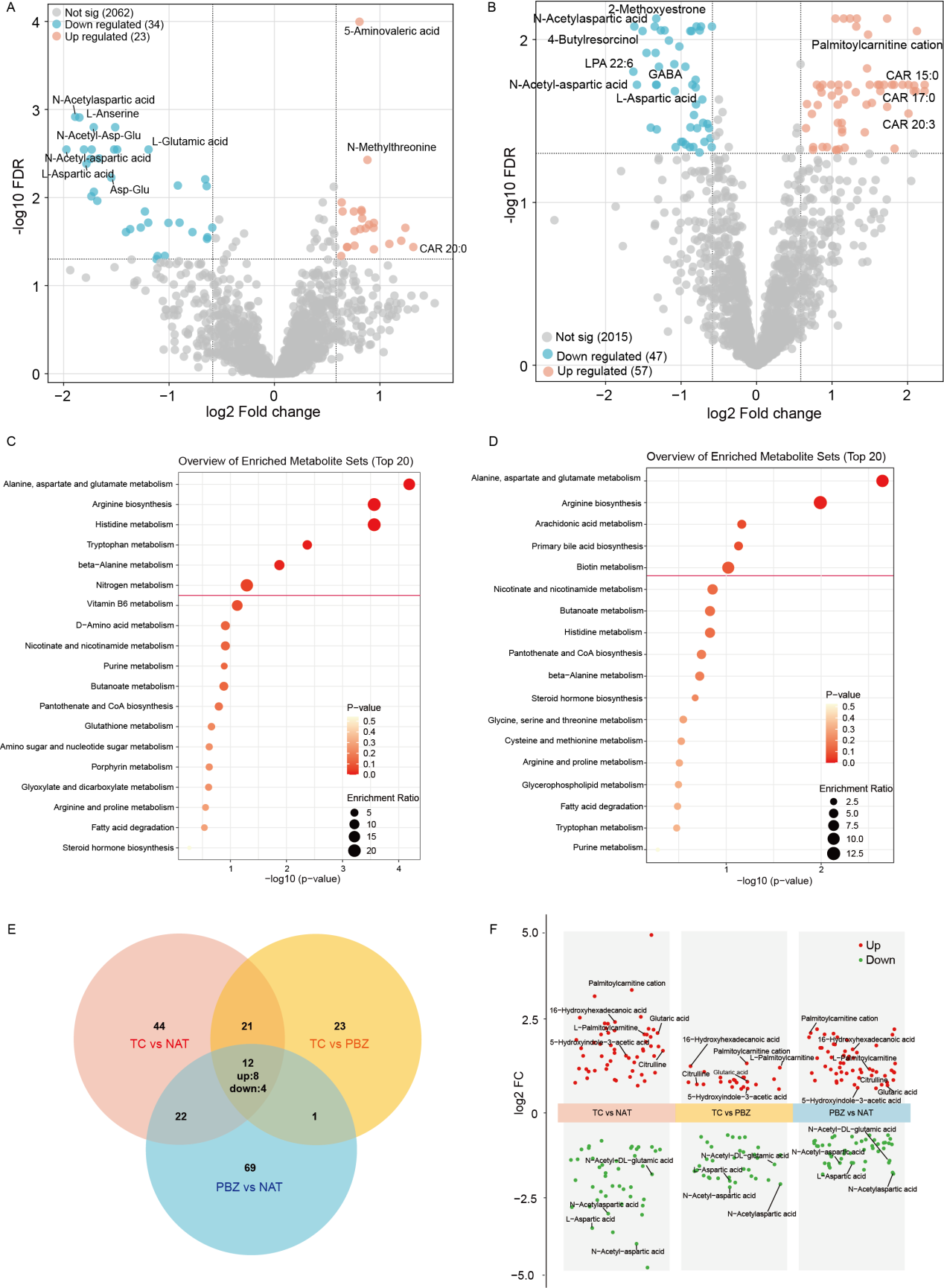


**Fig. S2.** Key metabolic transitions and biomarkers during spatial progression of glioma tissues. (A) Volcano plot analysis of TC samples versus PBZ samples in tumor tissues. *P*-values were determined by paired t-tests without correction. Differentially expressed metabolites are represented by blue dots (downregulated relative to PBZ samples) and red dots (upregulated relative to PBZ samples). Gray dots indicate no significant differences. (B) Volcano plot analysis of PBZ samples versus NAT samples in tumor tissues. *P*-values were determined by paired t-tests without correction. Differentially expressed metabolites are represented by blue dots (downregulated relative to NAT samples) and red dots (upregulated relative to NAT samples). Gray dots indicate no significant differences. (C) Top 20 most significantly altered metabolic pathways in TC tissues compared to PBZ tissues, with a red line delineating metabolic pathways with significant perturbations (*P* < 0.05). (D) Top 20 most significantly altered metabolic pathways in PBZ tissues compared to NAT tissues, with a red line delineating metabolic pathways with significant perturbations (*P* < 0.05). (E) Venn diagram illustrating the commonly differentially expressed metabolites among three comparisons: tumor cores versus adjacent normal brain tissues, tumor cores versus peritumoral brain tissues, and peritumoral brain tissues versus adjacent normal brain tissues. Significance thresholds: FDR < 0.05, FC > 1.5 or < 0.667. A total of 12 metabolites were commonly differentially expressed, with 8 upregulated and 4 downregulated. Detailed information of the commonly differentially expressed metabolites is listed in Supplementary Table 5. (F) Multiple volcano plots based on intergroup differences, showing the comparison of differentially expressed metabolites among three groups: tumor cores versus adjacent normal brain tissues, tumor cores versus peritumoral brain tissues, and peritumoral brain tissues versus adjacent normal brain tissues. Significance thresholds: metabolites with FDR < 0.05 and FC > 1.5 or < 0.667 are represented by solid circles, while metabolites with no significant differences are not shown.


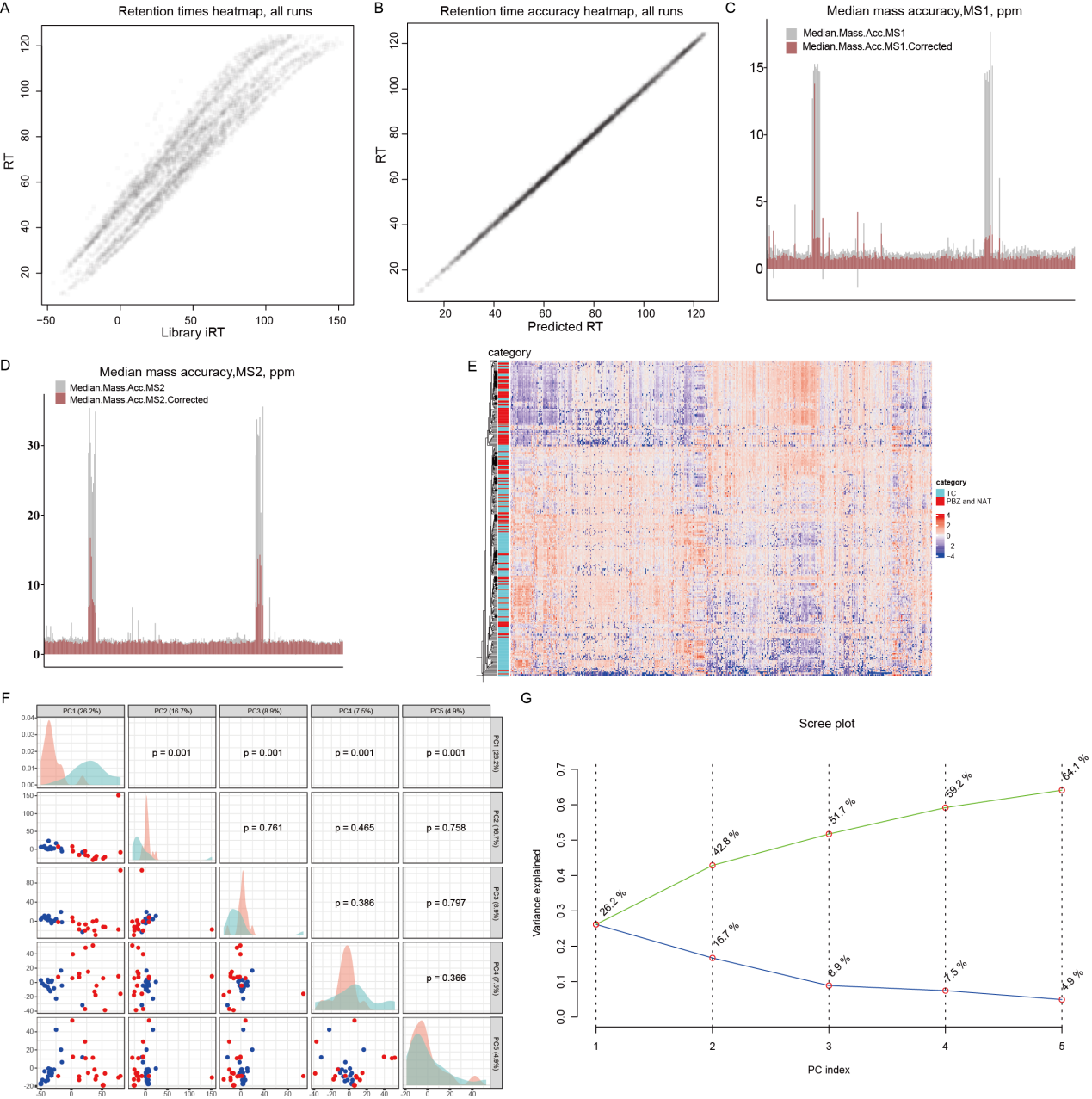


**Fig. S3.** LC-MS-based proteomic profiling of glioma tissues. (A) Retention time heatmap illustrating the correlation between observed retention times of samples and the library indexed retention time (iRT). The results indicate the stability of the experimental liquid chromatography system. (B) Retention time accuracy heatmap displaying the correlation of retention times among samples within batches. The results suggest better intra-batch stability of samples, demonstrating the stability of the separation equipment and methodology. (C and D)Mass errors of MS1 (C) and MS2 (D) for sample proteomic detection, with sample numbers on the x-axis and mass accuracy on the y-axis; gray represents uncorrected data, and red represents corrected data. The results show that the mass spectrometry data errors are within ±5 ppm, proving the data's good accuracy and stability. (E) Clustering heatmap of all quantifiable proteins, with sample clustering on the y-axis and protein clustering on the x-axis. (F) Pairwise comparison plots of the top five principal components from proteomic PCA analysis. (G) Scree plot showing the top five principal components of proteomic PCA analysis.


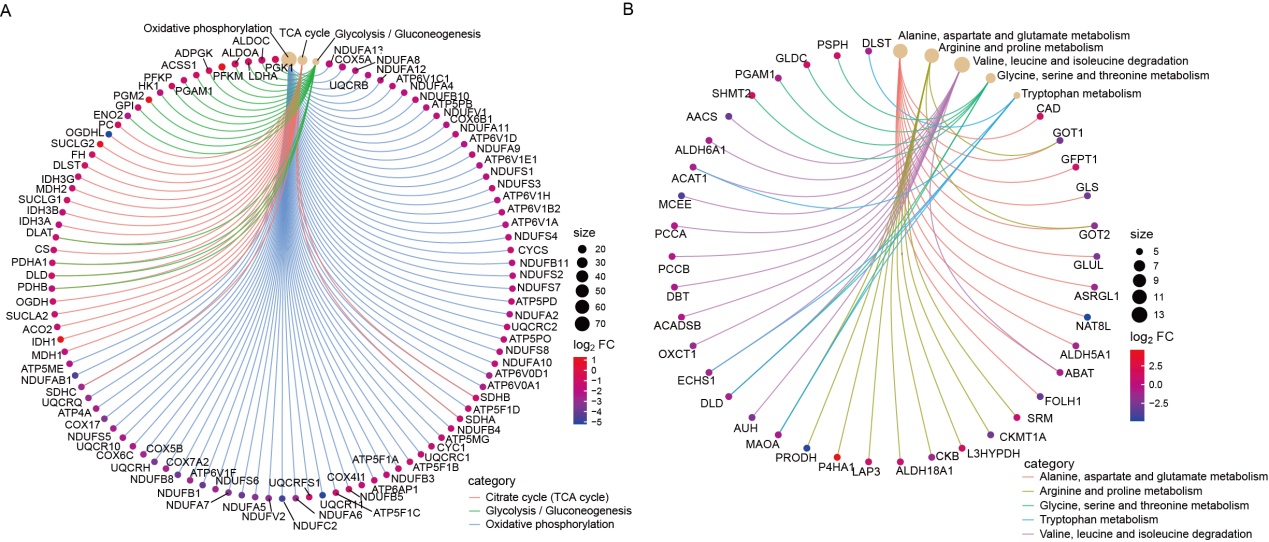


**Fig. S4.** Functional analysis of differentially expressed proteins in glioma tissues. (A and B) cnet plots display the expression levels of proteins involved in major energy metabolism (A) and amino acid metabolism (B) pathways.


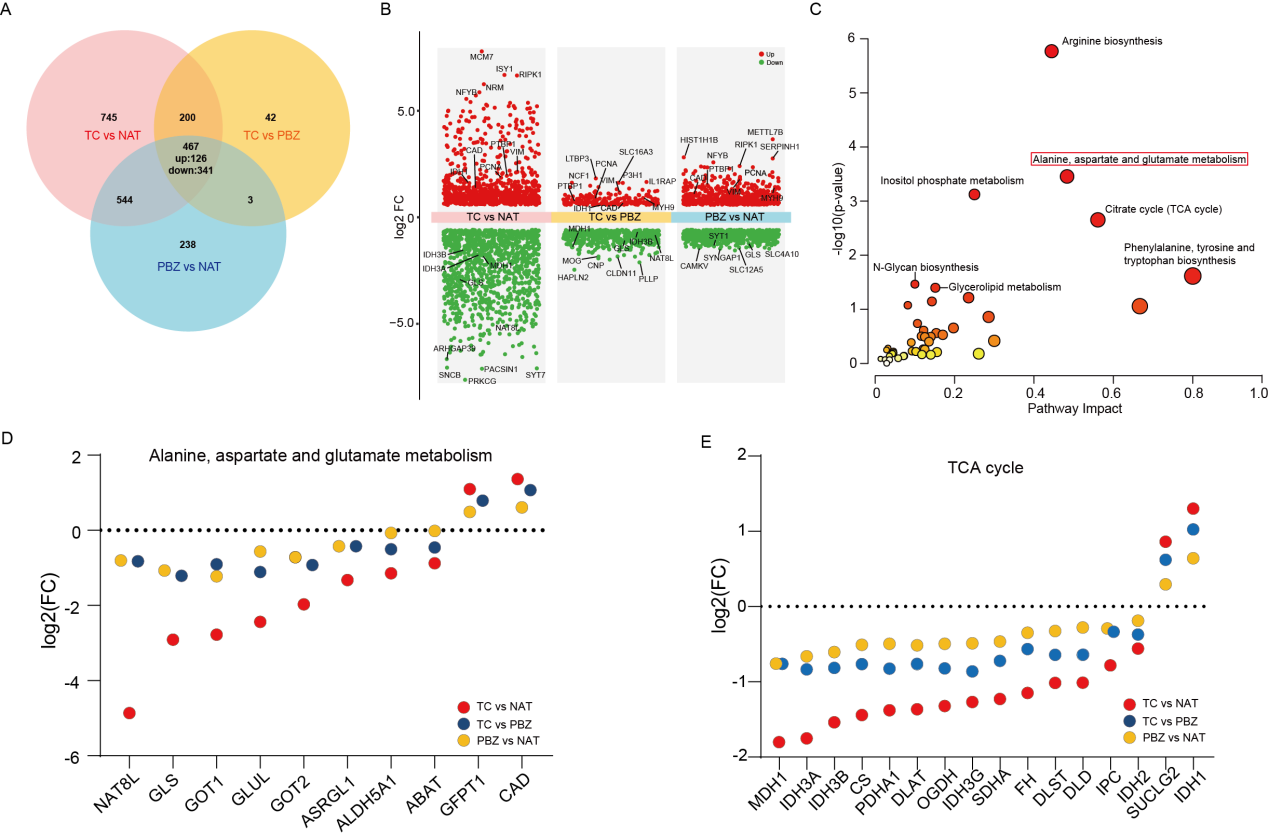


**Fig. S5.** Proteomic analysis in the spatial progression trajectory of glioma tissues. (A) Venn diagram illustrating the commonly differentially expressed proteins among three comparisons: tumor core versus adjacent normal brain tissue, tumor core versus peritumoral brain tissue, and peritumoral brain tissue versus adjacent normal brain tissue. Significance thresholds: FDR < 0.05, FC > 1.5 or < 0.667. (B) Multiple volcano plots based on intergroup differences, showing the comparison of differentially expressed proteins among three groups: tumor core versus adjacent normal brain tissue, tumor core versus peritumoral brain tissue, and peritumoral brain tissue versus adjacent normal brain tissue. Proteins meeting the significance thresholds (FDR < 0.05; FC > 1.5 or < 0.667) are represented by solid circles, while proteins with no significant differences are not shown. (C) Integrated pathway analysis of the commonly differentially expressed proteins (from Fig. S5B) and commonly differentially expressed metabolites (from Fig. S2E) during the spatial progression of glioma. (D and E) Expression differences of proteins involved in the ‘Alanine, aspartate, and glutamate metabolism’ (D) and ‘TCA Cycle’ (E) pathways during the spatial progression of glioma. Red dots represent tumor core tissue compared to paired adjacent normal brain tissue, blue dots represent tumor core tissue compared to paired peritumoral brain tissue, and yellow dots represent peritumoral brain tissue compared to adjacent normal brain tissue.


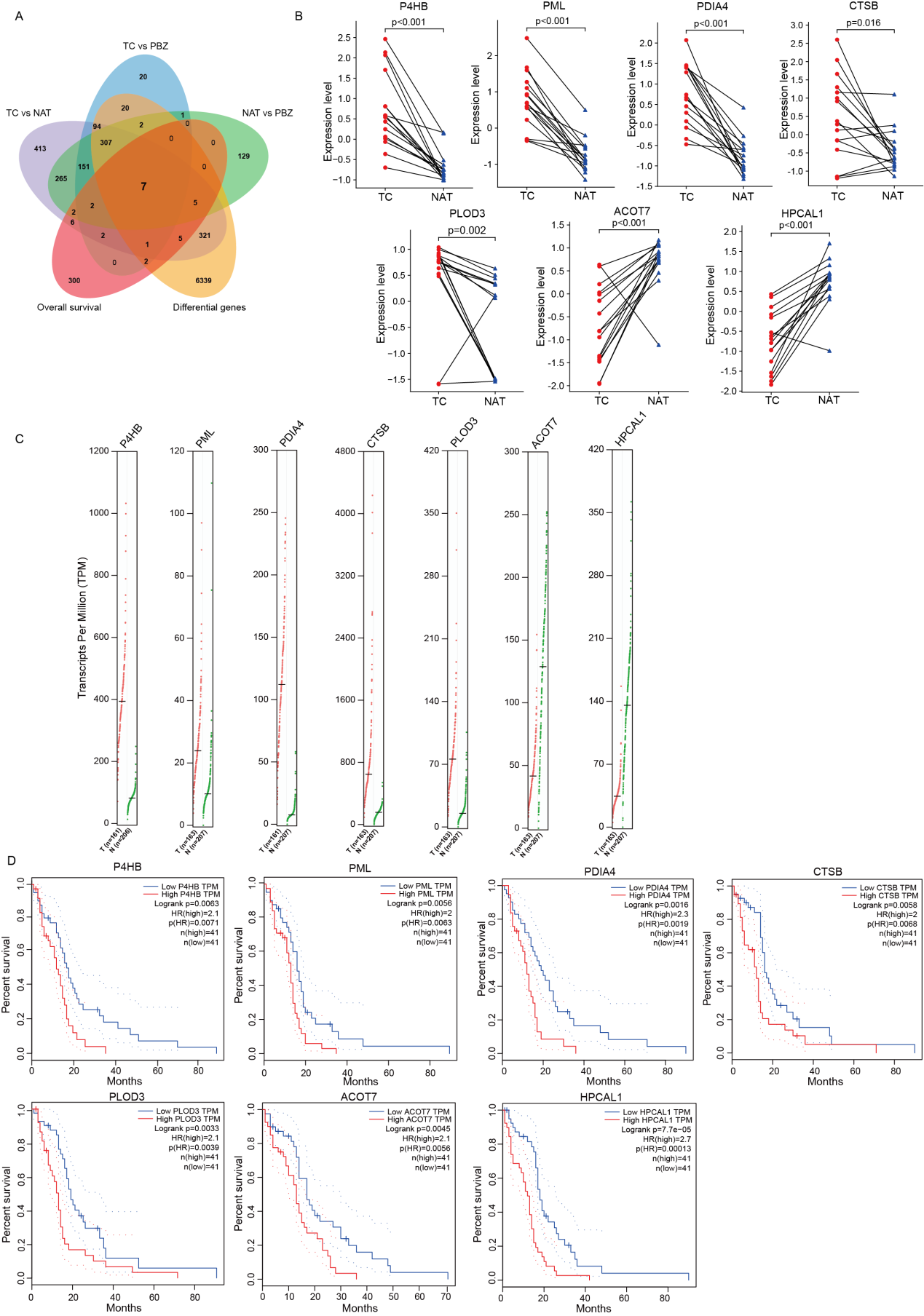


**Fig. S6.** Potential protein markers in glioma tissues. (A) Venn diagram illustrating the screening criteria for key proteins/genes: (1) differentially expressed during glioma progression; (2) significantly altered in tumor tissues versus normal brain tissues (analysis performed using GEPIA tool); and (3) significantly affecting overall patient survival in Kaplan-Meier analysis. A total of 7 proteins/genes were identified, with 5 showing significantly increased expression and 2 showing significantly decreased expression in glioma tissues. (B) Connectivity map displaying the expression levels of the 7 key proteins in tumor core tissues and adjacent normal brain tissues. P-values were determined by paired t-tests. (C) Comparative analysis of gene expression levels corresponding to the 7 key proteins in glioma tissues and normal brain tissues. Data were sourced from TCGA and GTEx databases, with analysis performed online using the GEPIA website (http://gepia.cancer-pku.cn/). (D) Kaplan-Meier survival curves demonstrating the impact of genes corresponding to the 7 key proteins on the overall survival of glioma patients.


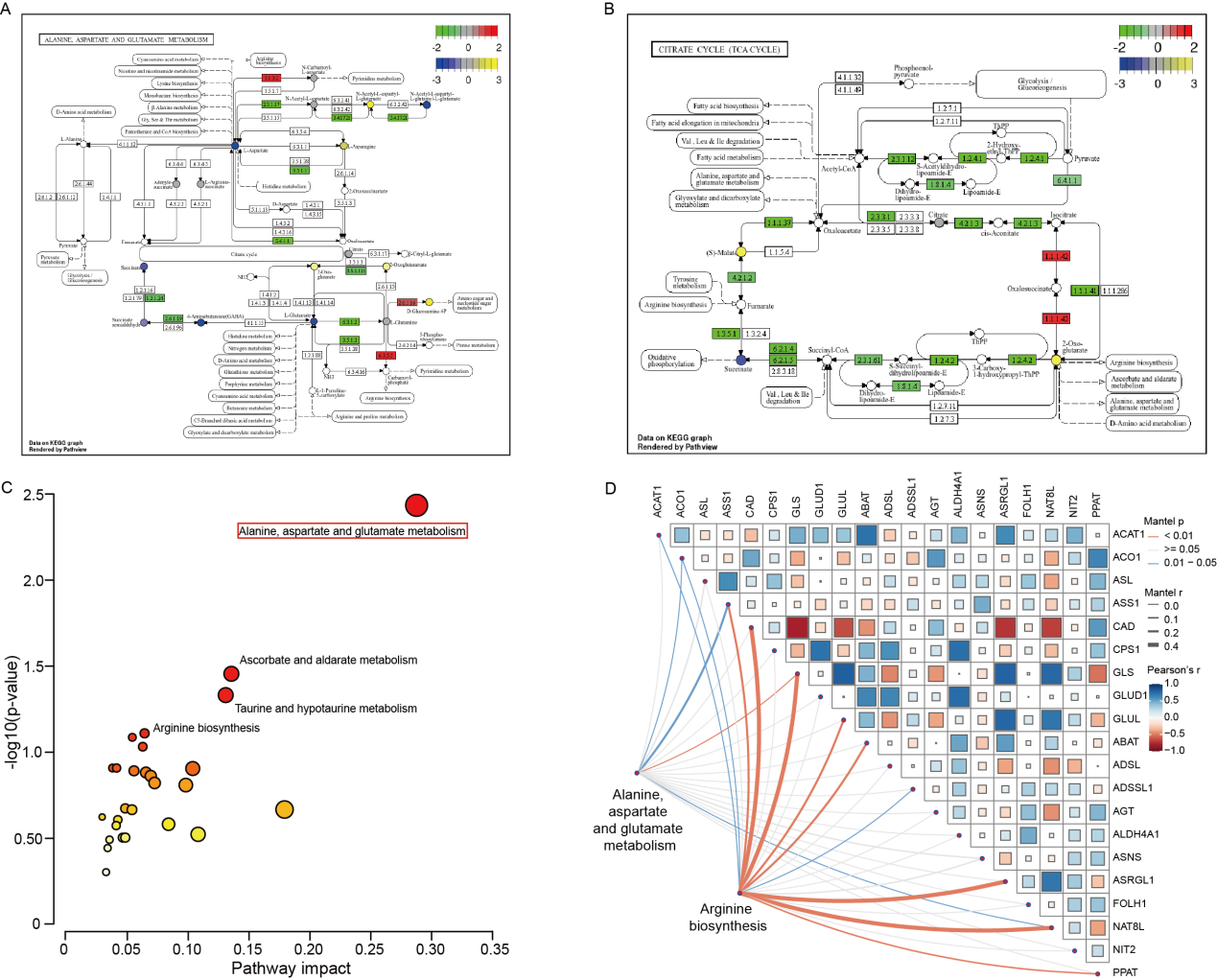


**Fig. S7.** Key pathways of metabolic reprogramming in glioma tissues. (A and B) Overview of the ‘Alanine, Aspartate, and Glutamate Metabolism’ (A) and ‘TCA Cycle’ (B) metabolic pathways in glioma tissues, including related metabolites (color representing log2 fold change values: green indicates downregulation, red indicates upregulation) and proteins (blue indicates downregulation; yellow indicates upregulation). (C) Integrated pathway analysis of the top 25 metabolites and proteins determined by the O2PLS-DA model, annotated using KEGG. (D) Mantel test analysis of the correlation between the metabolome and proteome of glioma tissues. Key metabolites in the ‘Alanine, aspartate, and glutamate metabolism’ or ‘Arginine biosynthesis’ pathway identified from tissue characteristics were compared with the proteome. The Mantel statistical results are provided in the right area of the chart. The network heatmap shows the correlation between the metabolome and proteome (the edge color indicates statistical significance (*P* values determined by two-tailed t-tests without adjustment for multiple comparisons), the edge width corresponds to the Mantel statistic for the respective distance correlation; the color gradient within the boxes indicates the Pearson correlation coefficient).


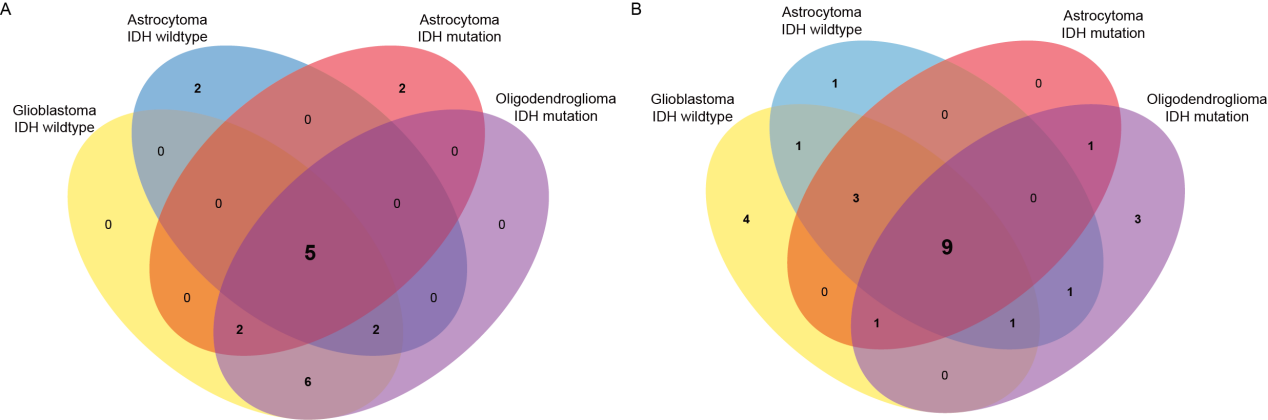


**Fig. S8.** Screening for common metabolic alteration pathways across adult glioma subtypes. (A and B) Venn diagrams illustrating the identification of significantly altered metabolic pathways commonly observed across all four adult glioma subtypes based on: (A) Metabolomics analysis; (B) Proteomics analysis.

**Fig. S9.** NMR-based plasma metabolomic profiles of glioma patients and healthy adults. (A and B) Overlapping NMR metabolic signal spectra (A) and signal dispersion spectra (B) of 20 aliquot quality control (QC) samples. (C) Correlation analysis between six shared clinical plasma metabolite biomarkers detected by NMR spectroscopy and conventional biochemical assays using univariate regression. The x-axis represents NMR-measured concentrations, while the y-axis represents concentrations measured by routine biochemical methods. (D) Statistical validation of the OPLS-DA model by permutation analysis (100 times). R² evaluates model goodness-of-fit, and Q² assesses predictive capability. (E) Decision tree plot generated by the random forest model for distinguishing adult glioma patients from healthy controls. Green lines represent glioma patients, whereas blue lines denote healthy individuals. (F) Top 15 metabolites with the highest mean decrease accuracy in the random forest model for differentiating glioma patients from healthy controls. Metabolites with a mean decrease accuracy exceeding 0.010 are highlighted to the right of the red dashed line.


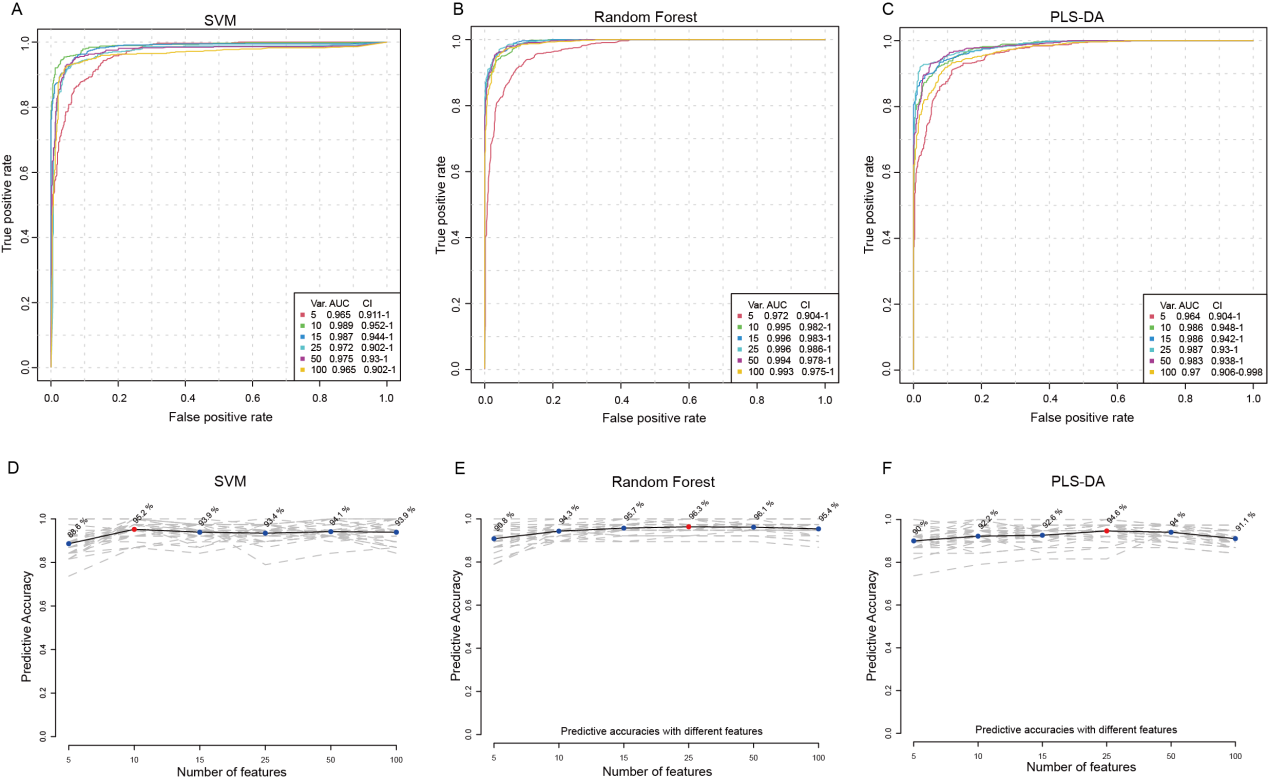


**Fig. S10.** Performance of machine learning models combining different plasma metabolite panels for discriminating glioma patients from healthy adults.

(A-C) ROC curves demonstrating the model performance of different metabolite combinations constructed using three machine learning algorithms: SVM (A), Random Forest (B), and PLS-DA (C). (D-F) Prediction accuracy of models constructed using different numbers of metabolite combinations with three machine learning algorithms: SVM (D), Random Forest (E), and PLS-DA (F).


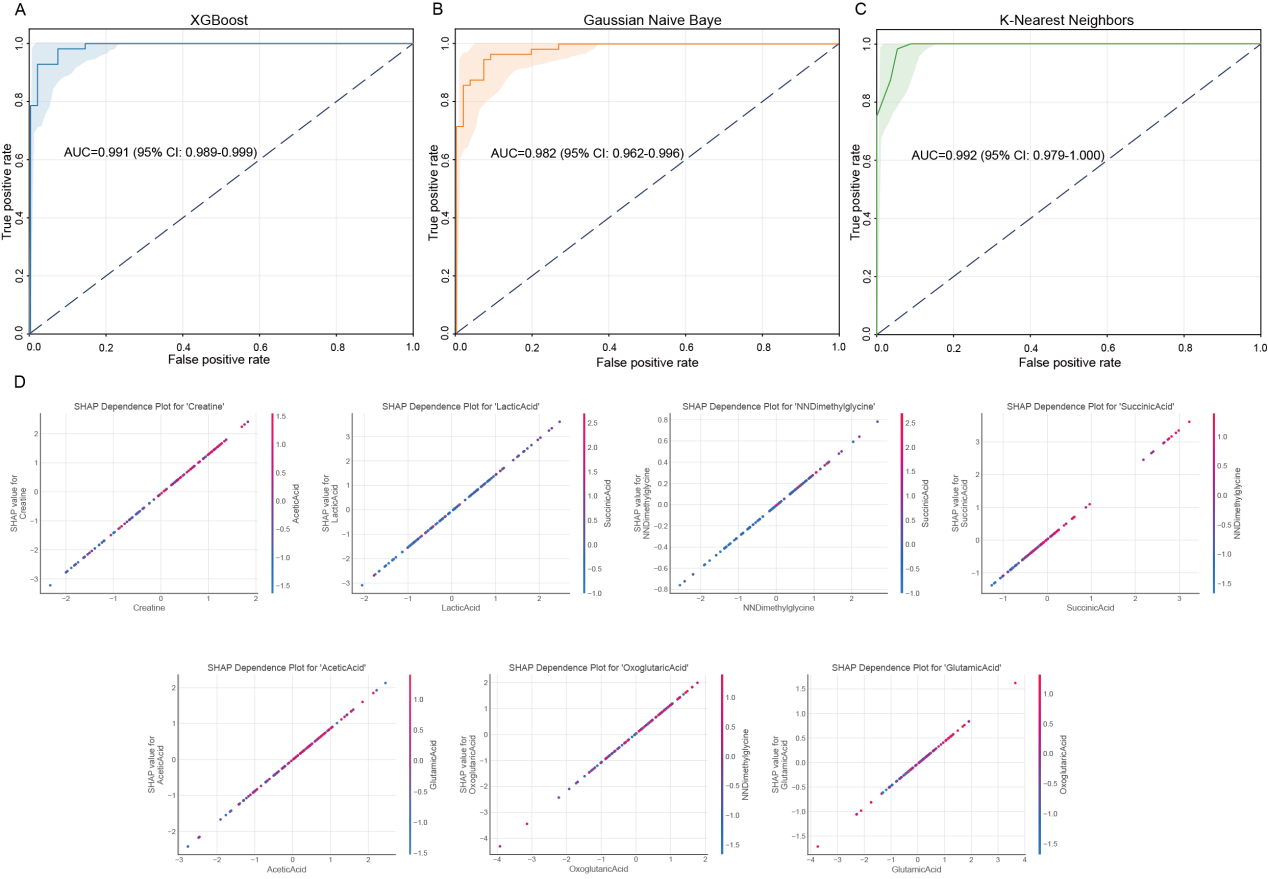


**Fig. S11.** Supplementary information for Clinical Diagnostic Models and SHAP Analysis. (A-C) ROC curves of clinical diagnostic models constructed using three machine learning algorithms: Extreme Gradient Boosting (XGBoost) (A), Gaussian Naive Bayes (B), and K-Nearest Neighbors (KNN) (C). (D) SHAP feature dependence plot.

**Table S1.** The demographic and clinical characteristics of glioma patients who provided tumor samples.

|  | All tumors,  N=122 (100%) | Glioblastoma,  N=50 (40.98%) | Astrocytoma,  N=42 (34.43%) | Oligodendroglioma,  N=24 (19.67%) | Mixed glioma,  N=6 (4.92%) |
| --- | --- | --- | --- | --- | --- |
| Gender; |  |  |  |  |  |
| Male | 81 (66.39%) | 36 (29.51%) | 30 (24.59%) | 12 (9.84%) | 3 (2.46%) |
| Female | 41 (33.61%) | 14 (11.48%) | 12 (9.84%) | 12 (9.84%) | 3 (2.46%) |
| Age at diagnosis; |  |  |  |  |  |
| Mean, years (SD) | 46.9 (14.1) | 46.5 (13.2) | 45.9 (13.1) | 47.3 (13.7) | 42.1 (8.5) |
| Range, years | 18-82 | 19-81 | 18-74 | 20-82 | 26-57 |
| <45 year old, n | 61 | 19 | 27 | 11 | 4 |
| 45-60 year old, n | 39 | 17 | 10 | 10 | 2 |
| 60-70 year old, n | 15 | 10 | 5 | 1 | 0 |
| >70 year old, n | 7 | 4 | 0 | 2 | 0 |
| WHO grades; |  |  |  |  |  |
| I | 3 | 0 | 2 | 0 | 1 |
| II | 45 | 0 | 23 | 17 | 5 |
| III | 20 | 0 | 13 | 7 | 0 |
| IV | 54 | 50 | 4 | 0 | 0 |
| IDH1 status; |  |  |  |  |  |
| IDH1-wildtype | 70 | 44 | 20 | 3 | 3 |
| IDH1-mutated | 52 | 6 | 22 | 21 | 3 |
| EGFR expression; |  |  |  |  |  |
| Negative | 8 | 4 | 3 | 1 | 0 |
| Positive | 107 | 43 | 35 | 23 | 6 |
| Not tested | 7 | 3 | 4 | 0 | 0 |
| MGMT protein expression; |  |  |  |  |  |
| low (0-30%), n | 34 | 8 | 15 | 10 | 1 |
| high (>30%), n | 88 | 42 | 27 | 14 | 5 |
| Not tested | 0 | 0 | 0 | 0 | 0 |
| 1p/19q; |  |  |  |  |  |
| codeleted | 24 | 1 | 0 | 12 | 0 |
| 1p deleted | 6 | 5 | 1 | 0 | 0 |
| 19q deleted | 5 | 3 | 2 | 0 | 0 |
| intact | 86 | 41 | 38 | 1 | 6 |
| Not tested | 1 | 0 | 1 | 0 | 0 |

**Table S2.** The demographic and clinical characteristics of glioma patients who provided plasma samples in training set.

|  | All tumors,  N=56 (100%) | Glioblastoma,  N=24 (42.86%) | Astrocytoma,  N=19 (33.93%) | Oligodendroglioma,  N=12 (21.43%) | Mixed glioma,  N=1 (1.79%) | Health Control, N=56 (100%) |
| --- | --- | --- | --- | --- | --- | --- |
| Gender; |  |  |  |  |  |  |
| Male | 34 (60.71%) | 14 (25%) | 10 (17.86%) | 10 (17.86%) | 1 (1.79%) | 33 |
| Female | 22 (39.29%) | 10 (17.86%) | 9 (16.07%) | 2 (3.57%) | 0 (0%) | 23 |
| Age at diagnosis; |  |  |  |  |  |  |
| Mean, years (SD) | 46.0 (13.53) | 47.43 (12.48) | 42.04 (11.42) | 46.57 (10.75) | 58 | 46.71 (13.18) |
| Range, years | 18-71 | 24-71 | 18-58 | 27-63 | 58 | 23-74 |
| <45 year old, n | 25 | 7 | 13 | 5 | 0 | 23 |
| 45-60 year old, n | 23 | 10 | 6 | 6 | 1 | 23 |
| 60-70 year old, n | 7 | 6 | 0 | 1 | 0 | 9 |
| >70 year old, n | 1 | 1 | 0 | 1 | 0 | 1 |
| WHO grades; |  |  |  |  |  |  |
| I | 1 | 0 | 1 | 0 | 0 | - |
| II | 20 | 0 | 10 | 9 | 1 | - |
| III | 8 | 0 | 5 | 3 | 0 | - |
| IV | 27 | 24 | 3 | 0 | 0 | - |
| IDH1 status; |  |  |  |  |  |  |
| IDH1-wildtype | 28 | 20 | 7 | 0 | 1 | - |
| IDH1-mutated | 28 | 4 | 12 | 12 | 0 | - |
| EGFR expression; |  |  |  |  |  |  |
| Negtive | 3 | 2 | 1 | 0 | 0 | - |
| Positive | 50 | 22 | 17 | 11 | 0 | - |
| Not tested | 3 | 0 | 1 | 1 | 1 | - |
| MGMT protein expression; |  |  |  |  |  |  |
| Low (0-30%), n | 13 | 4 | 5 | 4 | 0 | - |
| High (>30%), n | 41 | 20 | 13 | 8 | 0 | - |
| Not tested | 2 | 0 | 1 | 0 | 1 | - |
| 1p/19q; |  |  |  |  |  |  |
| Codeleted | 12 | 0 | 0 | 12 | 0 | - |
| 1p deleted | 3 | 2 | 1 | 0 | 0 | - |
| 19q deleted | 3 | 2 | 1 | 0 | 0 | - |
| Intact | 34 | 18 | 16 | 0 | 0 | - |
| Not tested | 4 | 2 | 1 | 0 | 1 | - |

**Table S3.** The demographic and clinical characteristics of glioma patients in adult glioma test set.

|  | All tumors,  N=26(100%) | Glioblastoma,  N=10 (38.46%) | Astrocytoma,  N=7 (26.92%) | Oligodendroglioma,  N=3 (11.54%) | Diffuse Midline Glioma,  N=4 (15.38%) | Ependymoma,  N=2 (7.68%) | Health Control, N=26 (100%) |
| --- | --- | --- | --- | --- | --- | --- | --- |
| Gender; |  |  |  |  |  |  |  |
| Male | 15 (57.69%) | 5 (19.23%) | 4 (15.38%) | 2 (7.69%) | 4 (15.38%) | 0 (0%) | 15 |
| Female | 11 (42.31%) | 5 (19.23%) | 3 (11.54%) | 1 (3.85%) | 0 (0%) | 2 (7.69%) | 11 |
| Age at diagnosis; |  |  |  |  |  |  |  |
| Mean, years (SD) | 47.42 (15.35) | 54.29 (9.96) | 49.63 (13.83) | 51.00 (3.65) | 35.43 (8.74) | 30 (4.42) | 46.62 (13.16) |
| Range, years | 18-74 | 40-72 | 25-74 | 45-55 | 18-49 | 24-36 | 26-73 |
| <45 year old, n | 12 | 2 | 5 | 0 | 3 | 2 | 12 |
| 45-60 year old, n | 8 | 4 | 0 | 3 | 1 | 0 | 11 |
| 60-70 year old, n | 4 | 3 | 1 | 0 | 0 | 0 | 1 |
| >70 year old, n | 2 | 1 | 1 | 0 | 0 | 0 | 2 |
| WHO grades; |  |  |  |  |  |  |  |
| I | 0 | 0 | 0 | 0 | 0 | 0 | - |
| II | 5 | 0 | 1 | 2 | 0 | 2 | - |
| III | 6 | 0 | 5 | 1 | 0 | 0 | - |
| IV | 15 | 10 | 1 | 0 | 4 | 0 | - |
| IDH1 status; |  |  |  |  |  |  |  |
| IDH1-wildtype | 20 | 10 | 4 | 0 | 4 | 2 | - |
| IDH1-mutated | 6 | 0 | 3 | 3 | 0 | 0 | - |
| EGFR expression; |  |  |  |  |  |  |  |
| Negtive | 2 | 1 | 0 | 0 | 1 | 0 | - |
| Positive | 23 | 9 | 7 | 3 | 3 | 1 | - |
| Not tested | 1 | 0 | 0 | 0 | 0 | 1 | - |
| MGMT protein expression; |  |  |  |  |  |  |  |
| Low (0-30%), n | 5 | 1 | 2 | 2 | 0 | 0 | - |
| High (>30%), n | 20 | 9 | 5 | 1 | 4 | 1 | - |
| Not tested | 1 | 0 | 0 | 0 | 0 | 0 | - |
| 1p/19q; |  |  |  |  |  |  |  |
| Codeleted | 3 | 0 | 0 | 3 | 0 | 0 | - |
| 1p deleted | 0 | 0 | 0 | 0 | 0 | 0 | - |
| 19q deleted | 0 | 0 | 0 | 0 | 0 | 0 | - |
| Intact | 20 | 9 | 7 | 0 | 3 | 1 | - |
| Not tested | 3 | 1 | 0 | 0 | 1 | 0 | - |

**Table S4.** The demographic and clinical characteristics of glioma patients in pediatric primary brain tumor test set.

|  | All tumors,  N=53(100%) | Glioblastoma,  N=5 (9.43%) | Astrocytoma,  N=20 (37.74%) | Medulloblastoma,  N=12 (22.64%) | Diffuse Midline Glioma,  N=8 (15.09%) | Ependymoma,  N=6 (11.32%) | Mixed glioma,  N=2 (3.77%) | Non-tumor Control, N=53 (100%) |
| --- | --- | --- | --- | --- | --- | --- | --- | --- |
| Gender; |  |  |  |  |  |  |  |  |
| Male | 33 (62.26%) | 2 (3.77%) | 12 (22.64%) | 9 (16.98%) | 4 (7.55%) | 5 (9.43%) | 1 (1.89%) | 28 |
| Female | 20 (37.73%) | 3 (5.66%) | 8 (15.09%) | 3 (5.66%) | 4 (7.55%) | 1 (1.89%) | 1 (1.89%) | 25 |
| Age at diagnosis; |  |  |  |  |  |  |  |  |
| Mean, years (SD) | 7.78 (4.26) | 9.50 (3.08) | 8.04 (4.14) | 7.61 (4.11) | 8.48 (0.96) | 6.36 (2.27) | 7.5 (1.50) | 11.51 (4.29) |
| Range, years | 0.5-17 | 5-17 | 2-17 | 0.5-17 | 7-10 | 3-10 | 5-10 | 2-17 |
| <10 year old, n | 38 | 4 | 12 | 10 | 8 | 5 | 1 | 17 |
| 10-17 year old, n | 15 | 1 | 8 | 2 | 0 | 1 | 1 | 36 |
| WHO grades; |  |  |  |  |  |  |  |  |
| I | 14 | 0 | 14 | 0 | 0 | 0 | 0 | - |
| II | 10 | 0 | 6 | 0 | 0 | 2 | 2 | - |
| III | 4 | 0 | 0 | 0 | 0 | 4 | 0 | - |
| IV | 25 | 5 | 0 | 12 | 8 | 0 | 0 | - |
|  |  |  |  |  |  |  |  |  |
| IDH1 status; |  |  |  |  |  |  |  |  |
| IDH1-wildtype | 50 | 5 | 20 | 11 | 8 | 4 | 2 | - |
| IDH1-mutated | 3 | 0 | 0 | 1 | 0 | 2 | 0 | - |
| EGFR expression; |  |  |  |  |  |  |  |  |
| Negtive | 17 | 3 | 11 | 3 | 0 | 0 | 0 | - |
| Positive | 32 | 2 | 8 | 7 | 8 | 5 | 2 | - |
| Not tested | 4 | 0 | 1 | 2 | 0 | 1 | 0 | - |
| MGMT protein expression; |  |  |  |  |  |  |  |  |
| Low (0-30%), n | 4 | 0 | 1 | 2 | 0 | 1 | 0 | - |
| High (>30%), n | 47 | 5 | 18 | 9 | 8 | 5 | 2 | - |
| Not tested | 2 | 0 | 1 | 1 | 0 | 0 | 0 | - |
| 1p/19q; |  |  |  |  |  |  |  |  |
| Codeleted | 0 | 0 | 0 | 0 | 0 | 0 | 0 | - |
| 1p deleted | 0 | 0 | 0 | 0 | 0 | 0 | 0 | - |
| 19q deleted | 1 | 0 | 0 | 0 | 0 | 1 | 0 | - |
| Intact | 41 | 4 | 20 | 6 | 7 | 3 | 2 | - |
| Not tested | 11 | 1 | 0 | 6 | 0 | 2 | 0 | - |

**Table S5.** The statistical data of 12 common differential metabolites during the spatial progression of glioma.

| Metabolism | Trends in Glioma | TC vs NAT | | TC vs PBZ | | PBZ vs NAT | |
| --- | --- | --- | --- | --- | --- | --- | --- |
|  |  | Fold Change | FDR | Fold Change | FDR | Fold Change | FDR |
| N-Acetyl-aspartic acid | ↓ | 0.11 | <0.001 | 0.25 | <0.001 | 0.40 | 0.0190 |
| L-Aspartic acid | ↓ | 0.12 | <0.001 | 0.29 | <0.001 | 0.40 | 0.0190 |
| N-Acetylaspartic acid | ↓ | 0.18 | 0.0022 | 0.27 | 0.0022 | 0.33 | 0.0083 |
| N-Acetyl-DL-glutamic acid | ↓ | 0.26 | 0.0031 | 0.44 | 0.0031 | 0.51 | 0.0425 |
| 5-Hydroxyindole-3-acetic acid | ↑ | 2.87 | 0.0022 | 1.57 | 0.0022 | 1.59 | 0.0249 |
| Citrulline | ↑ | 3.15 | 0.0264 | 1.69 | 0.0264 | 1.75 | 0.0252 |
| Lauric acid ethyl ester | ↑ | 3.40 | 0.0217 | 1.92 | 0.0217 | 2.78 | 0.0093 |
| L-Palmitoylcarnitine | ↑ | 4.25 | 0.0127 | 2.30 | 0.0127 | 2.49 | 0.0211 |
| Glutaric acid | ↑ | 4.35 | 0.0137 | 1.78 | 0.0137 | 1.57 | 0.0350 |
| cis-2-Decenoic acid | ↑ | 5.21 | 0.0056 | 1.92 | 0.0056 | 2.23 | 0.0233 |
| 16-Hydroxyhexadecanoic acid | ↑ | 5.34 | <0.001 | 2.37 | <0.001 | 3.32 | 0.0074 |
| Palmitoylcarnitine cation | ↑ | 9.63 | 0.0185 | 2.49 | 0.0185 | 4.36 | 0.0089 |

**Table S6.** Differential analysis of 7 key metabolites in pediatric primary brain tumors compared to AVMs.

| Metabolites | FDR | FC |
| --- | --- | --- |
| Creatine | <0.001 | 2.96 |
| Lactic acid | <0.001 | 2.44 |
| Succinic acid | <0.001 | 2.20 |
| N,N-dimethylglycine | <0.001 | 1.74 |
| 2-Oxoglutaric acid | <0.001 | 3.91 |
| Acetic acid | <0.001 | 4.01 |
| Glutamic acid | <0.001 | 1.86 |

**Table S7.** Expression level and discriminatory efficacy of the 7 metabolites in pancreatic cancer.

| **Metabolites** | **Glioma** | | | **Pancreatic cancer** | | |
| --- | --- | --- | --- | --- | --- | --- |
|  | **AUC** | **Fold Change** | **FDR** | **AUC** | **Fold Change** | **FDR** |
| creatine | 0.897 (0.827-0.950) | 2.71 | < 0.001 | 0.514 (0.420-0.607) | 1.338 | 0.689 |
| lactic acid | 0.874 (0.806-0.923) | 1.96 | < 0.001 | 0.898 (0.844-0.945) | 2.542 | < 0.001 |
| succinic acid | 0.856 (0.776-0.918) | 6.68 | < 0.001 | 0.838 (0.767-0.892) | 3.354 | < 0.001 |
| N,N-dimethylglycine | 0.855 (0.778-0.929) | 2.05 | < 0.001 | 0.769 (0.696-0.837) | 1.752 | < 0.001 |
| 2-oxoglutaric acid | 0.787 (0.694-0.874) | 3.81 | < 0.001 | 0.837 (0.766-0.900) | 7.034 | < 0.001 |
| acetic acid | 0.837 (0.756-0.902) | 2.66 | < 0.001 | 0.873 (0.814-0.925) | 2.491 | < 0.001 |
| glutamic acid | 0.784 (0.694-0.869) | 1.68 | < 0.001 | 0.854 (0.790-0.915) | 1.734 | < 0.001 |

**Table S8.** The effectiveness of plasma metabolites in differentiating between IDH mutation and wildtype gliomas.

| **Compound name** | **AUC** | **log2 FC** | ***P* value** |
| --- | --- | --- | --- |
| L-Cysteine | 0.950 | 0.559 | <0.001 |
| 2-Amino-4-oxopentanoic acid | 0.945 | 1.403 | <0.001 |
| 3-Hydroxy-L-proline | 0.942 | 1.367 | <0.001 |
| Malic Acid | 0.942 | 1.553 | <0.001 |
| L-Aspartate 4-semialdehyde | 0.930 | 1.079 | <0.001 |
| L-2-Amino-3-oxobutanoic acid | 0.927 | 1.039 | <0.001 |
| (3S)-3,6-Diaminohexanoic acid | 0.911 | 1.514 | <0.001 |
| Delta1-Piperideine-2-carboxylic acid | 0.882 | 0.979 | <0.001 |
| Xanthurenic acid | 0.875 | 1.185 | <0.001 |
| 6-Keto-prostaglandin Flalpha | 0.870 | -1.033 | <0.001 |
| lsomer 3 of Omithine | 0.856 | 1.310 | <0.001 |
| Cyclopropanecarboxylic acid | 0.841 | -0.606 | <0.001 |
| lsomer 1 of Histidine | 0.832 | -0.937 | <0.001 |
| 3,7-Dihydroxy-5-cholestanic acid | 0.828 | -0.684 | <0.001 |
| Hydroxyprolyl-Glutamine | 0.824 | -0.892 | <0.001 |
| L-Phosphinothricin | 0.812 | -0.812 | <0.001 |
| Phenylpyruvic acid | 0.811 | -0.435 | 0.0017 |
| lsomer 1 of L-Cysteinylglycine disulfide | 0.807 | -0.680 | <0.001 |
| 8-Demethyl-8-(methylamino)riboflavin | 0.802 | 0.304 | <0.001 |
| Spermidine | 0.797 | 0.780 | <0.001 |

**Table S9.** The effectiveness of plasma metabolites in differentiating between WHO grade III-IV and grade I-II gliomas.

| **Compound name** | **AUC** | **log2 FC** | ***P* value** |
| --- | --- | --- | --- |
| Kynurenine | 0.783 | -0.400 | <0.001 |
| (2S,5S)-trans-Carboxymethylproline | 0.771 | -0.422 | <0.001 |
| 2-Octenoic Acid | 0.770 | 2.277 | 0.0071 |
| Cholic acid-3-sulfate | 0.750 | -0.390 | <0.001 |
| Tyrosyl-Valine | 0.745 | -0.416 | 0.0014 |
| L-Tryptophan | 0.730 | -0.285 | 0.0023 |
| 5-Hydroxyindoleacetaldehyde O-glucuronide | 0.729 | -0.483 | 0.0014 |
| 13(S)-HPOT | 0.728 | -0.396 | 0.0013 |
| Valyl-Tyrosine | 0.728 | -0.360 | 0.0031 |
| 2-[(2-Aminoethylcarbamoyl)methyl] -2-hydroxybutanedioic acid | 0.727 | -0.411 | <0.001 |
| 8-Demethyl-8-(methylamino)riboflavin | 0.727 | -0.241 | 0.0023 |
| Adrenic acid | 0.726 | 0.576 | <0.001 |
| 3,4-Dihydroxyphenylvaleric acid | 0.724 | -0.262 | 0.0028 |
| Indolelactic Acid | 0.724 | -0.412 | 0.0050 |
| Glutamyl-Phenylalanine | 0.721 | 0.278 | 0.0018 |
| N-Acetylserotonin | 0.718 | -0.434 | 0.0012 |
| Allocystathionine | 0.714 | 0.504 | 0.0047 |
| Gentisate aldehyde 5-0-glucuronide | 0.710 | -0.287 | 0.0049 |
| Tyrosine O-glucuronide | 0.710 | -0.405 | 0.0023 |
| alpha-lsopropylmalic acid | 0.708 | 0.644 | <0.001 |
| 5-Chloro-1,2,4-trihydroxybenzene | 0.703 | 0.315 | 0.0048 |

**Table S10.** Data analysis of key plasma metabolites in healthy control participants and established clinical reference ranges.

| Metabolites(mmol/L) | Maximum | Minimum | Mean | Median | Standard deviation | Established clinical reference ranges |
| --- | --- | --- | --- | --- | --- | --- |
| Lactic acid | 4.8744 | 1.1037 | 2.4180 | 2.3849 | 0.7167 | 0.5-2.2 mmol/L |
| Glucose | 7.6704 | 3.2775 | 5.0207 | 4.8341 | 0.9111 | 3.9-6.1 mmol/L |
| Creatine | 0.0468 | 0.0044 | 0.0161 | 0.0145 | 0.0101 | 0.013-0.071 mmol/L |
| Creatinine | 0.0947 | 0.0404 | 0.0666 | 0.0656 | 0.0122 | 0.044-0.106 mmol/L |
| 3-Hydroxybutyric acid | 0.2228 | 0 | 0.0447 | 0.0282 | 0.0442 | 0-0.27 mmol/L |
| Pyruvic acid | 0.2882 | 0.0828 | 0.1569 | 0.1580 | 0.0404 | 0.03-0.10 mmol/L |
